# Initial Emergency Department Triage Narratives for Predicting Hospital Admission and Early Critical Outcomes: A Retrospective Chronological Split Study

**DOI:** 10.3390/jcm15218477

**Published:** 2026-10-30

**Authors:** Patryk Konieczka, Julia Żukowska-Karolak, Tomasz Kłosiewicz

**Affiliations:** 1 Department of Emergency Medicine, Poznan University of Medical Sciences, 60-806 Poznań, Poland; 2 Department of Medical Rescue, Poznan University of Medical Sciences, 60-806 Poznań, Poland

**Keywords:** emergency department, initial triage narrative, natural language processing, hospital admission, chronological split, calibration, intensive care, in-hospital mortality

## Abstract

**Background/Objectives:** We assessed whether initial triage narratives add predictive information beyond incompletely recorded structured data for recorded admission and early hospital outcomes. **Methods:** This single-center retrospective chronological split study included adults with Emergency Severity Index (ESI) levels 3–5 and available narratives. Penalized logistic models were developed on 51,735 visits (4852 admissions) from April to December 2025 and evaluated on 40,433 visits from January to June 2026. These data had previously been examined. Initial assessment selection was confirmed by the exporting investigator, but predictor timing could not be independently verified at record level. The primary outcome was recorded admission, including accepted external transfers; the secondary outcome was general intensive care unit (ICU) transfer or index-hospital death within seven days. **Results:** Evaluation included 3517 admissions (8.70%). Text increased admission area under the receiver operating characteristic curve (AUROC) from 0.763 to 0.835 (95% CI 0.828–0.841) and average precision from 0.246 to 0.340; the combined-model calibration slope was 1.083. The secondary outcome had 167 development events; evaluation included 40,190 visits and 123 events (0.306%); combined AUROC was 0.913 and average precision 0.055. At the development-selected 90% sensitivity target, secondary-model positive predictive value was 1.33%, with 204.5 flags per 1000 visits and 14 missed events. **Conclusions:** Narratives added information for local disposition beyond sparse structured documentation. These results do not establish prediction of physiological deterioration or an actionable alert policy. Independent temporal verification and external validation remain necessary. No prospective registration was obtained.

## 1. Introduction

Emergency department (ED) triage establishes urgency before diagnostic evaluation is complete, but patients assigned the same category can have different subsequent care requirements. Outcome-based electronic triage has identified important risk variation within Emergency Severity Index (ESI) level 3 [1]. Hospital admission and critical outcome prediction using routinely recorded triage information are established research tasks [2,3,4]. The contribution examined here is the incremental information in the initial Polish-language triage narrative alongside incompletely recorded structured triage data, assessed across a chronological split of a previously examined dataset.

Narratives can record presenting symptoms, their duration, functional changes and contextual information not represented in urgency categories or numeric fields. Prior studies have predicted ED disposition from nursing notes [5]. Arnaud et al. integrated structured information and text using a multi-input neural architecture [6], and Chang et al. combined these data types with evaluation at a second hospital [7]. A recent Journal of Clinical Medicine study also evaluated a triage hospitalization model at another center [8]. These studies establish the relevance of the question while highlighting the need to distinguish local predictive information from geographic transportability.

A prediction based on initial assessment must exclude information first becoming available after that assessment. Clinical NLP is vulnerable to label leakage when later diagnoses, treatment or disposition information enters the predictor [9]. Conversely, a referral diagnosis already known at arrival may be temporally eligible but can reflect a local care pathway rather than disease severity. Predictor timing, outcome construction and patient selection therefore require scrutiny alongside discrimination, consistent with prediction model risk-of-bias frameworks such as PROBAST + AI [10].

We investigated whether initial TOPSOR triage narratives added predictive information for recorded admission among adults assigned ESI levels 3–5. This primary outcome represents a local disposition decision. General intensive care unit (ICU) transfer and index-hospital death were analyzed separately within fixed horizons as early hospital outcomes; neither target establishes physiological deterioration after triage. We assessed calibration, clinically narrower groups, documentation changes and workload at exploratory operating points.

## 2. Materials and Methods

### 2.1. Setting, Population and Chronological Split

This single-center retrospective study used routine ED records from the University Clinical Hospital in Poznań, Poland for arrivals from 1 April 2025 through 30 June 2026. Eligible visits involved adults aged at least 18 years, ESI levels 3–5, a nonempty initial symptoms or circumstances field, recorded disposition and an ED departure time. Visits were the analytical unit; repeat visits were retained and visit-key uniqueness was checked. ESI 1–2 visits were excluded because they already indicate immediate or high-acuity assessment. The target population comprised lower-urgency patients, particularly the heterogeneous ESI 3 group.

**Figure 1:**
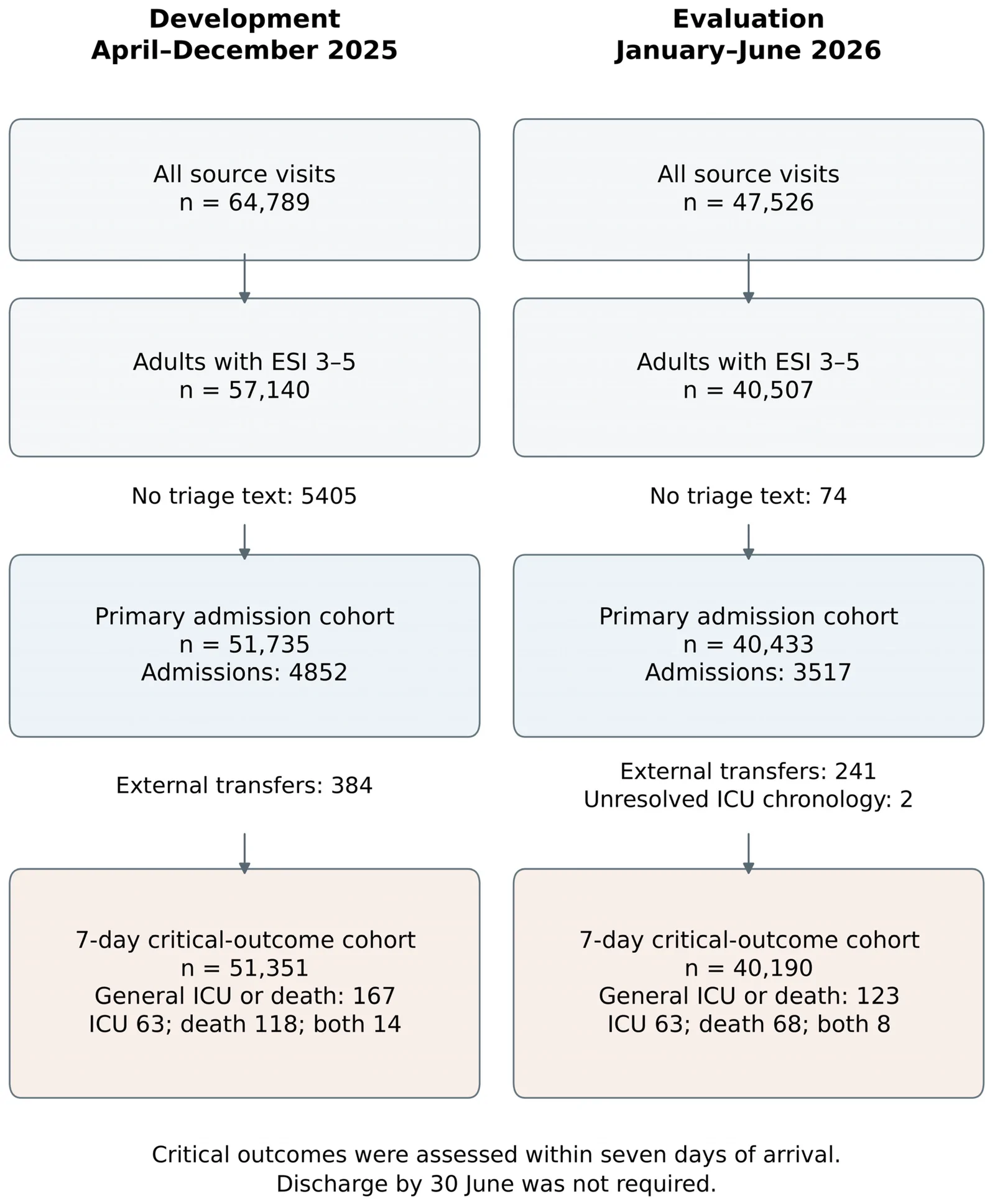
Study population and outcome-specific cohorts. Adult ESI 3–5 visits required initial narrative, known disposition and ED departure time. The hospital outcome cohorts exclude accepted external transfers. Two evaluation visits with unresolved ICU chronology are additionally excluded from ICU and composite outcomes; death-specific models include 40,192 visits. Hospital event sources cover the 30-day horizon for all included arrival dates.

Development used April–December 2025 and chronological evaluation January–June 2026. All preprocessing and coefficients were fitted using development data only. However, the dataset, including 2026, had previously been examined, and the outcome hierarchy, calendar partition and additional analyses were revised retrospectively. This was not an untouched confirmatory test or geographic external validation. Executed specifications and code, rather than a prospectively registered protocol, accompany a TRIPOD + AI reporting map [11]. Figure 1 summarizes cohort construction.

### 2.2. Initial Assessment, HIS Workflow and Predictor Provenance

Predictors were selected from the initial completed TOPSOR electronic triage card (KSM), within Poland’s nationally regulated ED triage framework [12,13,14,15]. In the study hospital, completion released the encounter for physician access in the hospital information system (HIS). The clinical text and completion timestamp then became non-editable; retriage created a separate record preserving the first assessment. The exporting investigator (P.K.) confirmed that this mechanism was unchanged during the study and that extraction selected the initial narrative and measurements, rather than retriage or physician documentation. This gate concerns electronic access, not the timing of verbal assessment.

Supplier specifications and operational guidance support separate initial and retriage records [16,17]. However, assessment identifiers, completion timestamps and field-level edit histories were absent from the research export. No independent record-level comparison with orders, consultations or disposition timestamps, or source-log audit, was performed. The workflow account supports the intended predictor boundary but does not independently verify the temporal integrity of every exported record. This limitation applies to both text and structured measurements.

Structured predictors were age, recorded sex, ambulance arrival, ESI, pulse, systolic and diastolic blood pressure, oxygen saturation, temperature, pain and respiratory rate. Text comprised symptoms and circumstances. Later consultations, investigation results, diagnoses, disposition and destination ward fields were excluded from predictors and used only for outcome construction or auditing.

### 2.3. Outcomes and Ascertainment

Recorded admission was defined by two disposition codes: internal inpatient admission or an agreed interhospital transfer accepted for inpatient treatment. Other dispositions, including ED death and non-routine departures, were negative for this administrative endpoint, without implying low clinical risk. Sensitivities excluded these departures or required internal admission lasting at least 24 h; external transfers and unresolved durations were excluded from the latter analysis.

The main secondary outcome was general ICU transfer or index-hospital death within seven days of arrival. Separate models examined ICU transfer within 72 h and death within seven or 30 days; ICU transfer within 24 h was counted descriptively. Dated ward movements captured direct and subsequent ICU transfers, and dated hospital death records included ED deaths. An expanded unit sensitivity added the mixed cardiac intensive care/internal medicine unit (OKW). Two evaluation visits with unresolved ICU dates before arrival were excluded from ICU/composite analyses but retained for death-specific analyses. Coding and chronology checks are detailed in Supplementary Methods.

Movement and death sources extended through 31 August 2026, covering the longest 30-day horizon for all arrivals without requiring discharge by June end. External transfers were excluded from hospital outcome models because receiving hospital outcomes were unavailable. Negative labels indicate no recorded index-hospital event within the horizon; they do not establish survival after discharge or elsewhere.

Outcomes were determined algorithmically without blinded clinical adjudication. Comfort-care or do-not-resuscitate status and validated referral status were unavailable. Linkage, event timing and discharge cutoff sensitivity are reported in Supplementary Tables S1–S3.

### 2.4. Cleaning and Missingness

Measurements outside broad technical plausibility ranges were set to missing (Table S5). Numeric inputs used development-set median replacement, missingness indicators and scaling; ESI was categorical. These procedures retain available documentation and do not recover unmeasured physiology.

All model comparisons used the same text-evaluable cohort. We summarized availability by month and outcome and described visits without narratives separately. Explanatory models separated core predictors (age, sex, ESI and ambulance arrival), measured values, missingness flags and text (Supplementary Methods and Tables S4–S6 and S10). The comparator therefore represents incompletely recorded structured data, not a comprehensive clinical triage assessment.

### 2.5. Text Processing and Prediction Models

The symptoms and circumstances fields were joined with a space, converted to lower case, stripped of Polish diacritics and reduced to single spaces. For example, the constructed phrase “BÓL W KLATCE” becomes “bol w klatce”. The model uses retained words and adjacent word pairs; because one-letter words are omitted, this example yields “bol”, “klatce” and “bol klatce”. Term frequency-inverse document frequency (TF-IDF) downweights widely occurring terms and normalizes each record’s vector. It does not independently interpret negation or historical symptoms.

Fixed admission/ward expressions and workflow words were removed. Separate L2-penalized logistic models used ESI only, structured predictors, text only, or structured predictors plus text; component and admission duration analyses used structured and combined models. No class weighting or oversampling was applied. Supplementary Methods provide the exact preprocessing, fixed model settings and masking lexicons.

Sensitivity refits used stricter masking, symptoms alone and an additional analyst-defined pathway lexicon specified after inspecting admission coefficients. These tests assess dependence on selected vocabulary, not source chronology. Contextual language encoders and blinded two-clinician term classification were not evaluated.

The 50 most positive and 50 most negative text coefficients per fitted endpoint, development document frequencies, structured coefficients and scaling are provided in Table S16 files. They describe conditional model associations rather than causal effects.

### 2.6. Performance, Uncertainty and Operating Points

We reported AUROC, average precision (AP), Brier score, calibration intercept and slope, calibration-in-the-large, observed-to-expected event ratio (O/E) and expected calibration error (ECE). Discrimination and probability calibration are distinct [18]; AP depends on prevalence [19], while class imbalance alone does not inflate AUROC [20]. Definitions and calculations are in Supplementary Methods.

Paired patient cluster bootstrap intervals used 500 resamples for main estimates and 300 for subgroups; unlinked visits formed singleton clusters. Intervals condition on fitted models. Subgroup AUROC/AP were suppressed with fewer than ten events or non-events. ESI 3 and internally admitted patient results appear in the main text.

Five-fold stratified patient-grouped cross-validation refitted all preprocessing and model components within development folds. Pooled out-of-fold predictions selected thresholds targeting 80%, 90% and 95% sensitivity, applied unchanged to 2026. We report positive predictive value (PPV), flags per 1000 visits and missed events alongside discrimination; the 90% target illustrates workload and is not an alert recommendation. All eligible available visits were used without a prospective sample size calculation [21].

### 2.7. Documentation Changes and Additional Analyses

Post hoc analyses examined alternate development periods (excluding July–August or using December only), evaluation restrictions and documentation variability. Supplementary Methods specify every variant and monthly text length and vocabulary drift calculations; Tables S9, S12 and S13 report their results. These analyses did not select a replacement model using evaluation performance.

Conditional decision curves [22] explored hypothetical earlier bed coordination for admission risk and prioritized reassessment for the seven-day outcome. Actions and threshold ranges were analyst-defined; no intervention or decision impact was tested. Full assumptions and pointwise uncertainty appear in Supplementary Methods, Table S14 and Figure S1.

An offline single-record runtime benchmark excluded the HIS pathway and concurrent clinical workload (Supplementary Methods and Table S15). It was not a production system evaluation.

## 3. Results

### 3.1. Cohort and Documentation

The study period source contained 112,315 visits: 64,789 in development and 47,526 in evaluation. The admission cohorts comprised 51,735 and 40,433 text-evaluable adult ESI 3–5 visits, with 4852 and 3517 admissions. Table 1 compares the cohorts. Evaluation admissions included 3276 internal admissions and 241 accepted external transfers.

Narrative was absent in 5405 of 57,140 adult ESI 3–5 development visits (9.46%) and 74 of 40,507 evaluation visits (0.18%). The development gap was concentrated in July and August. After cleaning, pulse was available in 31.2% and 47.8% of the respective cohorts, and respiratory rate in 4.2% and 6.0%. Patient linkage was unavailable for 22,926 development visits (44.3%) and five evaluation visits. Monthly completeness and the text-empty population are described in Supplementary Tables S4–S6.

### 3.2. Recorded Admission

**Figure 2:**
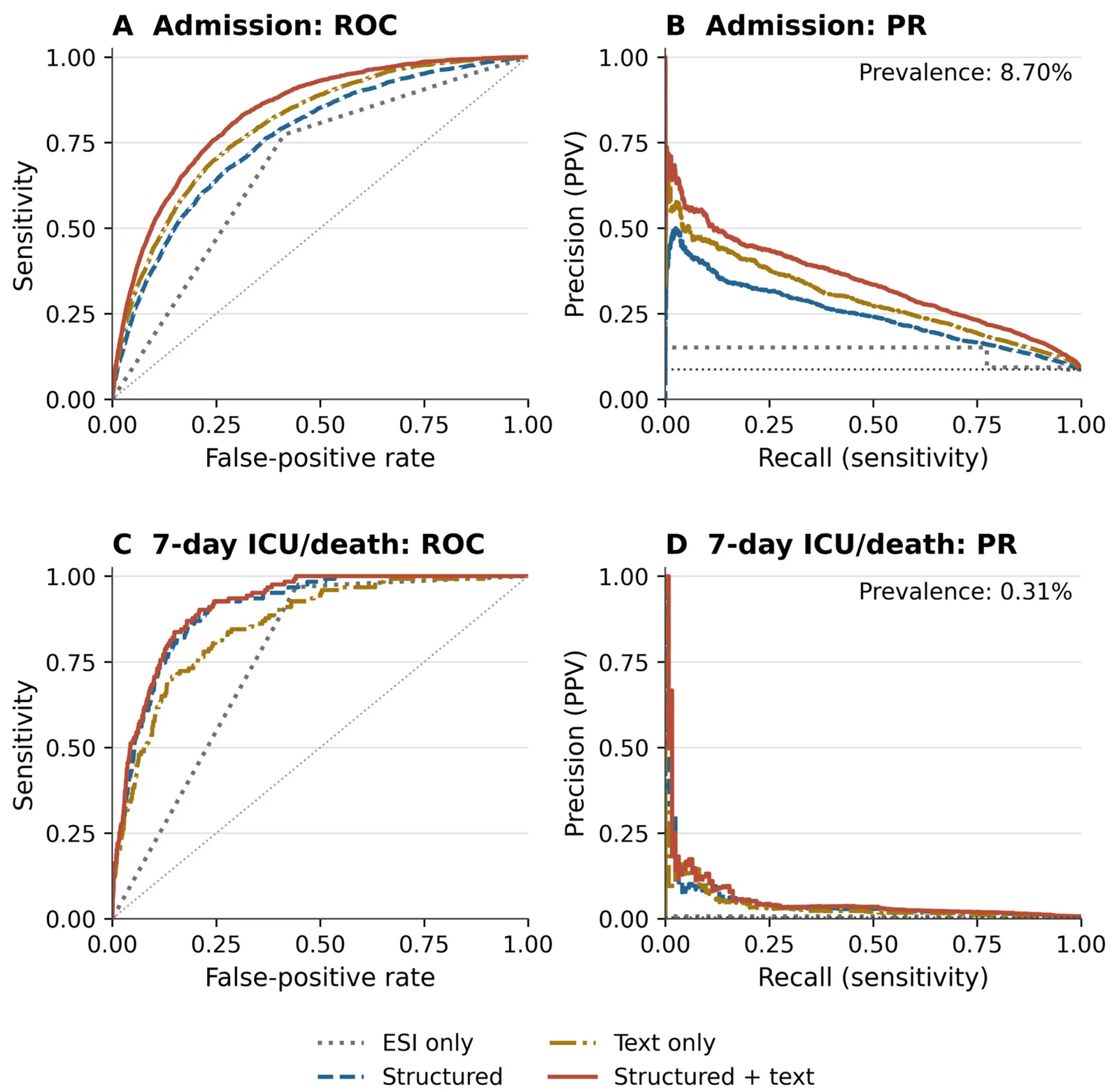
Receiver operating characteristic (ROC) and precision–recall curves for recorded admission and seven-day general ICU transfer or index-hospital death. Dotted baselines indicate chance ROC discrimination or observed event prevalence. Models within an endpoint use identical evaluation visits; absolute precision is not comparable across endpoints without considering prevalence.

Adding narrative increased admission AUROC from 0.763 to 0.835 (0.828–0.841) and AP from 0.246 to 0.340 (0.325–0.357). Paired increments were 0.072 (95% CI 0.065–0.077) for AUROC and 0.094 (95% CI 0.083–0.106) for AP. Table 2 reports all four models; Figure 2 shows discrimination curves.

**Figure 3:**
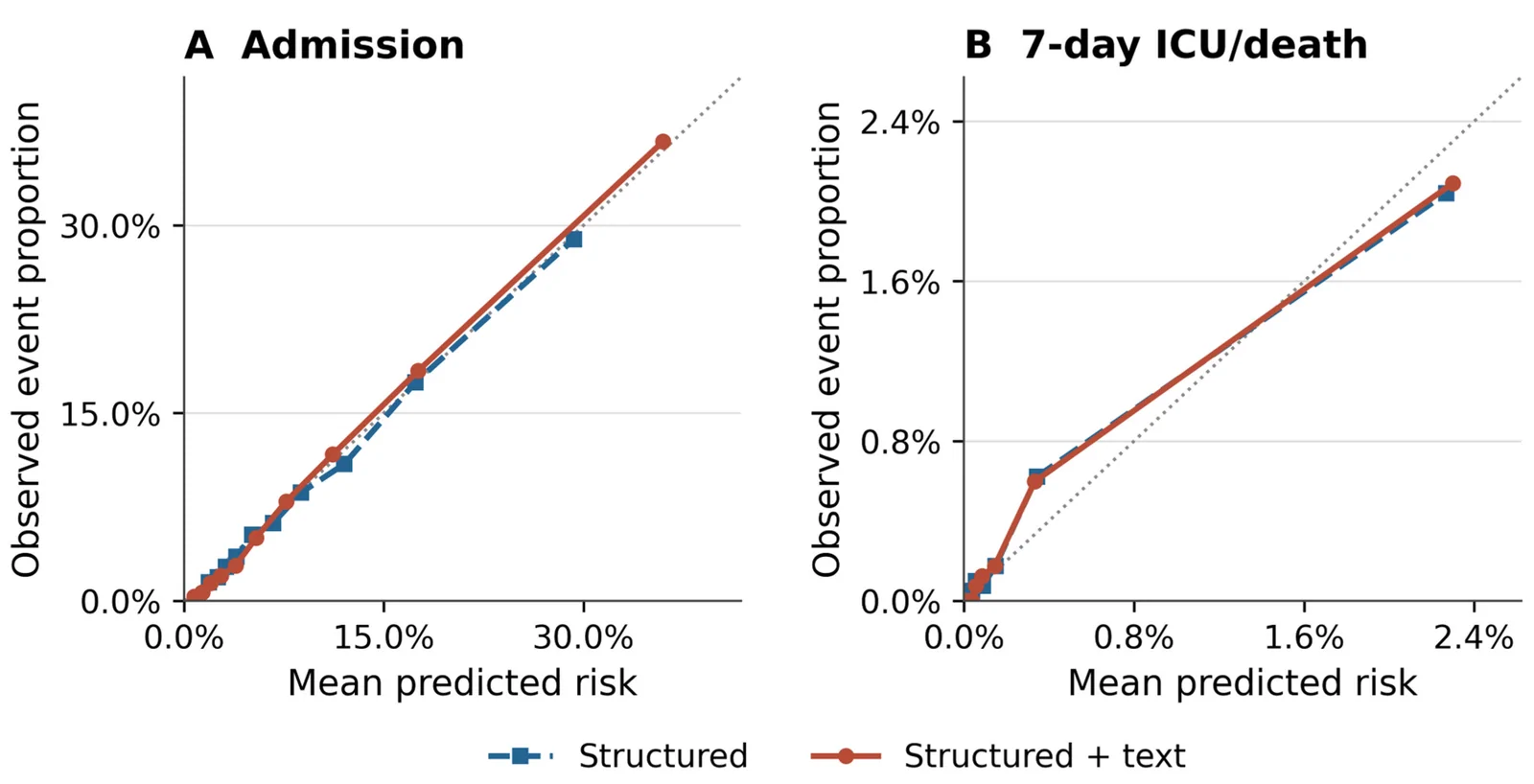
Calibration of structured and combined models in the chronological evaluation cohort. Points are observed and mean predicted risks in tie-preserving probability groups; the diagonal denotes agreement. The rare outcome risk scale differs from the admission scale. Group-level calibration does not guarantee accurate individual predictions.

The combined admission model had calibration intercept 0.125, slope 1.083, O/E 0.982 and ECE 0.00621. Brier score improved from 0.07242 to 0.06696, while ECE increased from 0.00429 (Figure 3).

### 3.3. Fixed-Horizon Outcomes and Clinically Narrower Groups

The seven-day composite occurred in 167 of 51,351 development visits and 123 of 40,190 evaluation visits. Evaluation events comprised 63 general ICU transfers and 68 index-hospital deaths, with eight visits experiencing both within seven days. There were 27 ICU transfers within 24 h, 43 within 72 h, and 156 in-hospital deaths within 30 days. The combined seven-day model achieved AUROC 0.913 (0.897–0.930) and AP 0.055 (0.031–0.096); paired increments over structured data were 0.009 (95% CI 0.006–0.014) and 0.013 (95% CI 0.002–0.022), respectively. Its calibration slope was 0.874, O/E 1.014 and ECE 0.00066. Table 3 presents component-specific and subgroup performance.

For ICU transfer within 72 h, the AP increment was 0.00016 (95% CI −0.00033 to 0.00088), providing no clear evidence of improved precision for this component.

Within ESI 3, the seven-day combined-model AUROC was 0.828 (0.795–0.861) with 119 events; among internally admitted patients it was 0.750 (0.715–0.788) with 117 events and a calibration slope of 0.515. Median arrival-to-event time was 18.5 h (IQR 7.3–34.4) for ICU transfers within 72 h and 71.0 h (35.7–109.2) for deaths within seven days (Table S2).

### 3.4. Operating Points and Calendar Performance

At the illustrated target (Table 4), the admission combined model had PPV 17.25%, 447.7 flags per 1000 visits and 394 missed admissions. The seven-day model flagged 8217 visits, with 109 true positives, 8108 false positives and 14 missed events. PPV was 1.33%, corresponding to 74.4 false positive flags per true positive.

**Figure 4:**
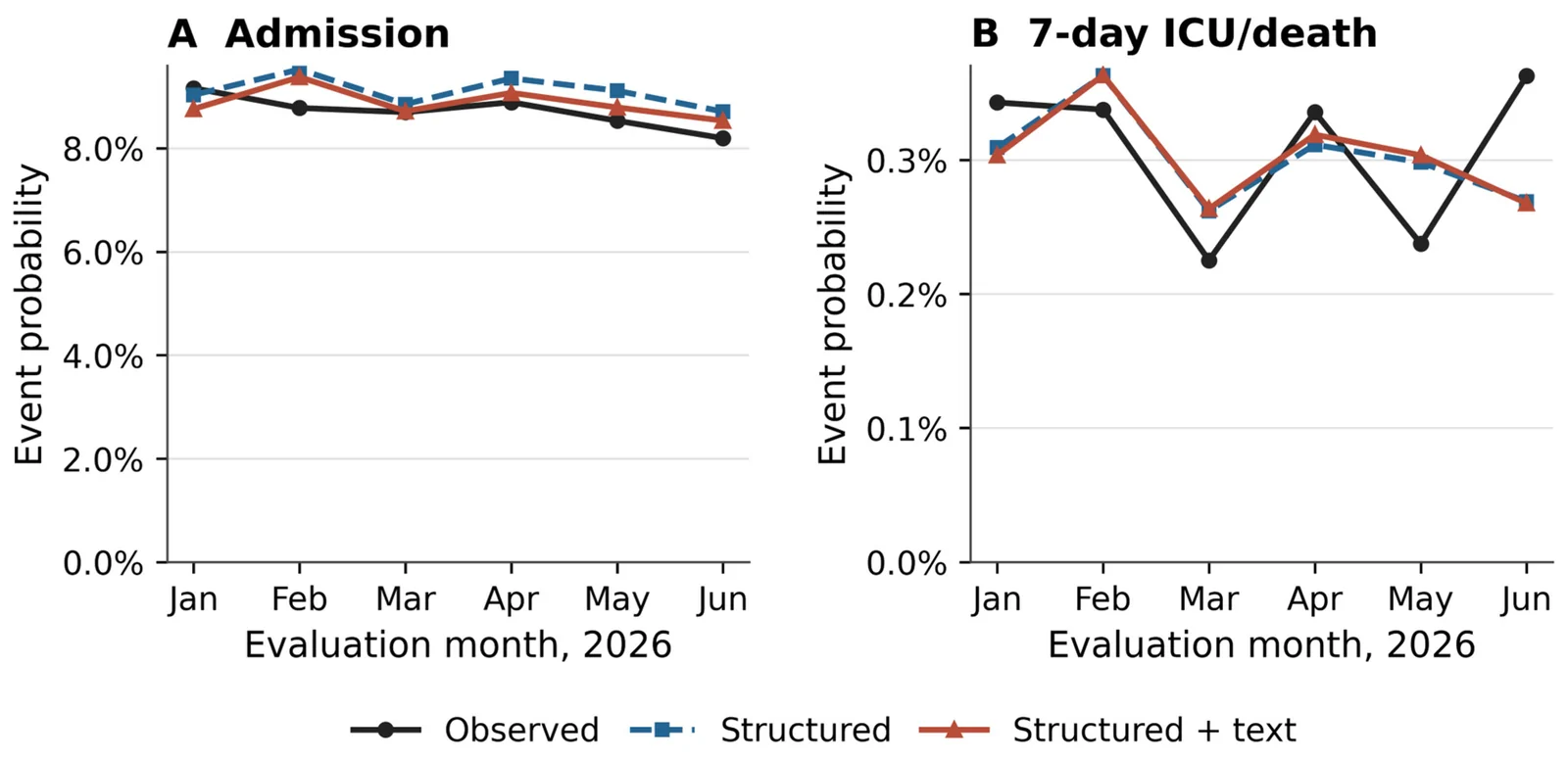
Monthly observed event frequency and mean combined-model predicted probability. Cohorts use fixed-horizon event ascertainment through the hospital sources ending in August. Differences reflect calendar variation in outcomes, case mix, documentation and model calibration; the figure does not identify a causal source of drift.

Monthly combined-model admission AUROC ranged from 0.824 to 0.848 and O/E from 0.936 to 1.046. Seven-day event counts ranged from 15 to 26 per month and O/E from 0.783 to 1.355 (Figure 4).

### 3.5. Robustness and Documentation Dependence

Core-plus-text models omitted all seven measured fields and their missingness indicators, achieving admission AUROC/AP 0.832/0.332 and seven-day values 0.912/0.039. Table 5 compares the recorded value, missingness and narrative contributions.

Admission AUROC was 0.836 after strict phrase removal and 0.836 using symptoms alone; corresponding seven-day values were both 0.913. Excluding non-routine departures and ED deaths yielded admission AUROC/AP 0.834/0.347 and seven-day values 0.911/0.055. Expanded pathway masking and the remaining sensitivities are reported in Table S9.

Excluding July–August from development yielded admission/seven-day AUROC 0.832/0.915; December-only development yielded 0.801/0.872. The latter used 6530 visits with 492 admissions and 6466 visits with 20 seven-day events, respectively (Table S9).

Internal admission lasting at least 24 inpatient hours occurred in 3102 of 40,145 evaluation visits; combined AUROC was 0.842 (0.836–0.848) and AP 0.321. Including OKW in the seven-day composite increased events to 286, with AUROC 0.899 (0.883–0.911) and AP 0.061 (Table S9).

Median narrative length fell from 124 characters in November to 55 in December 2025 and remained 47–54 during evaluation. Vocabulary divergence increased concurrently (Table S13; Figure S2). The cause of these documentation changes could not be established from the exports.

### 3.6. Model Interpretation, Development Diagnostics and Conditional Utility

Positive admission terms included “okulistyki” (ophthalmology), “okulistyczny” (ophthalmic), “ustalone” (arranged/established), “ortopedii” (orthopedics) and “crp”; negative terms included “bol” (pain), “uraz” (injury), “zaczerwienienie” (redness) and “rana” (wound). These include clinical descriptions and specialty pathway vocabulary. Table 6 shows representative coefficients; Table S16 files provide all selected terms and document frequencies.

Admission apparent and grouped out-of-fold AUROC/AP were 0.868/0.434 and 0.826/0.335; seven-day values were 0.947/0.157 and 0.918/0.087. The combined models contained 9104 and 9018 fitted predictors, respectively. Table S12 compares these diagnostics with chronological performance.

Combined-minus-structured net benefit per 1000 visits was 10.704 (95% CI 9.287 to 12.042) for admission at 10% risk, and −0.008 (95% CI −0.159 to 0.119) for the seven-day outcome at 0.5% risk. Figure S1 and Table S14 provide all hypothetical thresholds; these are conditional calculations, not observed intervention benefits.

## 4. Discussion

### 4.1. Interpretation and Contribution

Initial narratives improved prediction of recorded admission beyond incompletely recorded structured triage data: AUROC increased from 0.763 to 0.835 and AP from 0.246 to 0.340. This finding does not establish added value over a comprehensive physiological assessment. Core-plus-text comparisons support a narrative contribution without measured fields or their missingness flags but cannot resolve the effects of sparse and changing documentation.

Recorded admission represents a local administrative and clinical disposition decision, influenced by bed availability, specialty pathways, referral arrangements and clinician practice as well as patient need. Accepted external transfers identify known inpatient destinations. Even admission lasting at least 24 h remains an operational proxy, not adjudicated medical necessity. Predicting that decision is therefore distinct from identifying physiological deterioration, and a model score does not determine whether admission or discharge is appropriate.

Text-based disposition prediction and combining structured and unstructured data are established approaches [2,5,6,7]. Our contribution is the paired assessment of initial Polish triage narratives in an ESI 3–5 population with sparse measured fields, across a retrospective chronological split, with explicit source workflow evidence and fixed-horizon hospital outcomes. Studies with external hospital cohorts address geographic transportability that this analysis cannot establish [7,8]. Differences in language, patient spectrum, documentation and endpoint definitions prevent ranking these studies by their headline AUROCs.

Narrative can convey symptom burden and context alongside referral pathways and prior diagnoses already known at arrival. Temporally eligible information may still limit clinical interpretation and transferability. Coefficients make selected associations inspectable but do not establish causal importance. The transparent lexical reference was not compared with contextual language models.

### 4.2. Clinical Meaning and Practical Performance

The seven-day composite measures recorded ICU transfer or index-hospital death, not adjudicated worsening from the initial clinical state. A fixed horizon and separate component analyses improve its definition but do not remove this distinction. Severe illness or an end-of-life trajectory may already be evident at triage, and ICU transfer depends on decisions and capacity. The lower discrimination within ESI 3 and admitted patients shows the importance of patient spectrum when interpreting whole-cohort AUROC.

The seven-day outcome occurred in 0.306% of evaluation visits. Its AUROC of 0.913 coexisted with AP 0.055 and, at the illustrated target, PPV 1.33%, 204.5 flags per 1000 visits and 14 missed events. The 8108 false positive flags for 109 detected events imply substantial workload. Absence of the endpoint does not prove reassessment would be unnecessary, but its benefit was not tested. The decision curve increment at the 0.5% threshold included zero. These results show predictive information rather than clinically actionable risk stratification; no threshold is recommended for disposition or escalation.

Clinical use would require independent validation in a different setting, prospective assessment of a specified response, acceptable workload, calibration monitoring and evidence of patient benefit. Unavailable or non-finalized input should lead to ordinary clinical assessment without a model output. Offline runtime does not establish HIS integration or concurrent ED performance; prehospital use was not investigated.

### 4.3. Limitations

Predictor timing could not be independently verified at record level. The implemented TOPSOR workflow, exporting investigator’s confirmation and technical documentation support initial assessment selection, but assessment identifiers, completion timestamps and edit histories were absent from the research dataset. The HIS release gate and separate retriage records do not replace a source-log audit of exported rows. This evidence gap does not establish leakage but prevents a definitive conclusion that every narrative and measurement preceded subsequent clinical information. Text-masking analyses cannot resolve it.

The structured comparator contained very sparse measurements, especially respiratory rate and temperature. Median replacement does not reconstruct an unrecorded physiological assessment, and missingness may itself reflect documentation behavior. The comparison therefore concerns information available in incomplete structured records. Excluding visits without narratives also selects a text-evaluable population.

This single-center analysis used previously examined data. Chronological separation preserved the fitting boundary but did not create an independent confirmatory evaluation; endpoint revisions and robustness analyses were retrospective. Changes in text, measurement availability and patient linkage may reflect documentation or extraction conditions, whose causes remain unresolved. An unchanged TOPSOR locking mechanism does not imply unchanged documentation completeness. December-only development also uses a smaller, seasonally restricted sample. Generalizability to other hospitals, languages, children, ESI 1–2 visits or encounters lacking text is unknown.

Hospital sources do not capture post-discharge deaths or receiving-hospital outcomes. The fixed-horizon labels concern recorded index-hospital events; comfort-care or do-not-resuscitate stratification, blinded outcome adjudication and validated referral status analysis were unavailable. ICU definitions follow available ward codes rather than adjudicated critical care requirements.

Missing development patient identifiers leave unrecognized repeat patient overlap possible despite grouped cross-validation and newly observed-patient analyses. Bootstrap intervals condition on fitted models; small component and monthly event counts limit precision. High-dimensional penalized models can overfit, and no comprehensive fairness assessment or blinded two-clinician term classification was performed. Future independent studies should predefine outcomes and analyses, retain assessment identifiers and timestamps, and test transportability before broader implementation.

## 5. Conclusions

Initial narratives added predictive information for local admission decisions beyond incompletely recorded structured triage data. The seven-day hospital outcome model showed limited precision and substantial false positive workload, supporting further study rather than clinically actionable risk stratification. Neither endpoint establishes physiological deterioration. Unverified record-level predictor timing, previously examined evaluation data and the single-center setting require independent verification and external validation before clinical use.

## Figures and Tables

**Table 1:** Characteristics and recorded measurement availability.

Characteristic	Development: Apr–Dec 2025	Evaluation: Jan–Jun 2026
Eligible visits	51,735	40,433
Age, median (IQR), years	46 (32–68)	46 (31–66)
Male, n (%)	24,917 (48.2%)	19,550 (48.4%)
Ambulance arrival, n (%)	5301 (10.2%)	3967 (9.8%)
ESI 3, n (%)	22,541 (43.6%)	17,972 (44.4%)
ESI 4, n (%)	23,549 (45.5%)	18,220 (45.1%)
ESI 5, n (%)	5645 (10.9%)	4241 (10.5%)
Recorded admission, n (%)	4852 (9.4%)	3517 (8.7%)
Pulse available after cleaning	31.2%	47.8%
Systolic pressure available after cleaning	39.9%	47.6%
Diastolic pressure available after cleaning	38.8%	47.6%
Oxygen saturation available after cleaning	34.3%	54.4%
Temperature available after cleaning	12.5%	12.6%
Pain score available after cleaning	12.0%	35.3%
Respiratory rate available after cleaning	4.2%	6.0%

IQR: interquartile range. Availability describes recorded values remaining after quality control, not whether a measurement was clinically performed.

**Table 2:** Chronological evaluation performance.

Outcome/Model	AUROC (95% CI)	AP (95% CI)	Brier	ECE	O/E
Admission ESI only	0.681 (0.674–0.689)	0.138 (0.133–0.144)	0.07619	0.00856	0.917
Admission Structured	0.763 (0.755–0.771)	0.246 (0.234–0.259)	0.07242	0.00429	0.958
Admission Text only	0.800 (0.793–0.807)	0.292 (0.278–0.309)	0.07005	0.01216	1.000
Admission Structured + text	0.835 (0.828–0.841)	0.340 (0.325–0.357)	0.06696	0.00621	0.982
ICU/death ≤ 7 days ESI only	0.764 (0.746–0.777)	0.007 (0.005–0.008)	0.00304	0.00025	0.924
ICU/death ≤ 7 days Structured	0.904 (0.884–0.923)	0.042 (0.026–0.083)	0.00302	0.00066	1.019
ICU/death ≤ 7 days Text only	0.858 (0.828–0.887)	0.034 (0.020–0.066)	0.00303	0.00258	0.928
ICU/death ≤ 7 days Structured + text	0.913 (0.897–0.930)	0.055 (0.031–0.096)	0.00300	0.00066	1.014

Admission: 40,433 visits, 3517 events. Seven-day ICU/death: 40,190 visits, 123 events. AP is average precision, not a trapezoidal precision–recall area. O/E is observed events divided by summed predicted probabilities. Full calibration and additional intervals appear in Table S7.

**Table 3:** Components and clinically narrower evaluation groups.

Outcome/Group/Model	Events/Visits	AUROC (95% CI)	AP (95% CI)
General ICU ≤ 72 h Structured	43/40,190	0.846 (0.782–0.901)	0.012 (0.004–0.056)
General ICU ≤ 72 h Structured + text	43/40,190	0.851 (0.793–0.903)	0.012 (0.005–0.056)
Hospital death ≤ 7 days Structured	68/40,192	0.941 (0.926–0.956)	0.037 (0.021–0.085)
Hospital death ≤ 7 days Structured + text	68/40,192	0.948 (0.934–0.961)	0.045 (0.027–0.089)
Hospital death ≤ 30 days Structured	156/40,192	0.917 (0.895–0.938)	0.053 (0.039–0.080)
Hospital death ≤ 30 days Structured + text	156/40,192	0.930 (0.911–0.948)	0.069 (0.050–0.098)
Admission; ESI 3 Structured	2719/17,972	0.685 (0.676–0.697)	0.281 (0.268–0.297)
Admission; ESI 3 Structured + text	2719/17,972	0.762 (0.753–0.774)	0.372 (0.356–0.392)
ICU/death ≤ 7 days; ESI 3 Structured	119/17,808	0.813 (0.777–0.848)	0.047 (0.027–0.088)
ICU/death ≤ 7 days; ESI 3 Structured + text	119/17,808	0.828 (0.795–0.861)	0.058 (0.032–0.104)
ICU/death ≤ 7 days; admitted Structured	117/3276	0.742 (0.704–0.780)	0.112 (0.079–0.164)
ICU/death ≤ 7 days; admitted Structured + text	117/3276	0.750 (0.715–0.788)	0.116 (0.080–0.168)

Component models were fitted separately. ESI 3 and internally admitted rows evaluate the original endpoint model within the stated group, without subgroup refitting. Eventual admission is a post-triage subgroup definition, not a deployable eligibility rule. Death-specific cohorts include the two visits excluded for unresolved ICU chronology. Other subgroup estimates are in Table S8; discrimination is suppressed below ten events or non-events.

**Table 4:** Development-selected 90% sensitivity target applied to 2026.

Outcome/Model	Threshold	Sensitivity	PPV	Flags/1000	False Positives/Missed
Admission Structured	0.04185	90.2%	12.54%	625.6	22,123/345
Admission Structured + text	0.05422	88.8%	17.25%	447.7	14,977/394
ICU/death ≤ 7 days Structured	0.00199	87.0%	1.30%	204.3	8102/16
ICU/death ≤ 7 days Structured + text	0.00194	88.6%	1.33%	204.5	8108/14

Thresholds were selected from five-fold grouped development out-of-fold predictions. They are descriptive workload examples, not recommended clinical actions. Complete confusion matrices, specificity and additional sensitivity targets appear in Table S11.

**Table 5:** Explanatory comparison of predictor sets.

Predictors	Admission AUROC	Admission AP	7-Day AUROC	7-Day AP
Core	0.752	0.222	0.894	0.022
Core + missingness	0.754	0.224	0.891	0.021
Core + imputed measurements	0.762	0.245	0.908	0.044
Structured	0.763	0.246	0.904	0.042
Core + text	0.832	0.332	0.912	0.039
Core + missingness + text	0.832	0.333	0.909	0.035
Structured + text	0.835	0.340	0.913	0.055

Core: age, recorded sex, ESI and ambulance arrival. Measured fields: pulse, systolic and diastolic pressure, oxygen saturation, temperature, pain and respiratory rate. All variants use the same endpoint-specific cohorts. These post hoc comparisons are not a model selection procedure. Intervals and paired differences appear in Table S10.

**Table 6:** Four most positive and four most negative lexical coefficients per main model.

Outcome	TERM/Bigram	Coefficient	Development Documents
Admission	na	2.783	23,021
Admission	okulistyki	2.251	98
Admission	okulistyczny	2.157	165
Admission	sm	2.157	93
Admission	bol	−2.966	18,495
Admission	uraz	−1.994	8730
Admission	zaczerwienienie	−1.930	1097
Admission	palca	−1.898	1652
ICU/death ≤ 7 days	wymioty	0.874	2158
ICU/death ≤ 7 days	ca	0.712	371
ICU/death ≤ 7 days	odwodnienie	0.690	46
ICU/death ≤ 7 days	kontaktu	0.646	177
ICU/death ≤ 7 days	glowy	−0.581	5451
ICU/death ≤ 7 days	uraz	−0.529	8709
ICU/death ≤ 7 days	bol	−0.467	18,371
ICU/death ≤ 7 days	uraz glowy	−0.389	1208

Terms are shown after normalization. Coefficients are per unit TF-IDF in the combined model, conditional on all other predictors. Document frequency is the number of development narratives containing the term under the fitted analyzer. Unigrams and bigrams can be correlated; coefficients are not causal effects. All 100 selected terms per fitted endpoint and structured coefficients appear in Table S16.

## Data Availability

Patient-level data and original narratives are not publicly available because they contain sensitive health information under institutional control. Access requests may be considered by the institution subject to authorization and applicable data protection requirements. Code, aggregate outputs and selected model coefficients are provided as Supplementary Materials. Full model objects and vocabularies are not distributed.

## Supplementary Materials

The following supporting information can be downloaded at: https://www.mdpi.com/article/10.3390/jcm15218477/s1, Table S1. Cohort accounting and endpoint-specific denominators; Table S1 continued. Events over eligible visits; Table S2. Time from ED arrival to recorded events; Table S3. Sensitivity to a hospital-discharge-date restriction; Table S4. Monthly source availability among adult ESI 3–5 visits; Table S5. Technical cleaning ranges and measurement availability; Table S6. Missing measurements by recorded admission; Table S6 continued. Visits with and without narrative text; Table S7. Full endpoint performance and calibration; Table S7 continued. Probability calibration; Table S8. Admission performance within clinical groups; Table S8 continued. ICU/death within 7 days performance within clinical groups; Table S9. Admission sensitivity analyses; Table S9 continued. ICU/death within 7 days sensitivity analyses; Table S9 continued. Expanded pathway masking and alternative endpoint definitions; Table S10. Fixed predictor-set and missingness comparisons; Table S11. Admission operating points; Table S11 continued. ICU/death within 7 days operating points; Table S11 continued. Confusion counts and false-positive burden; Table S12. Apparent, grouped out-of-fold and chronological performance; Table S13. Monthly narrative length and vocabulary drift; Table S14. Admission conditional decision analysis; Table S14 continued. ICU/death within 7 days conditional decision analysis; Table S15. Offline single-record prediction runtime; Table S16. Coefficient files provided with the supplement; Figure S1. Exploratory decision curves for hypothetical actions. Top panels show net benefit per 1000 visits for the structured model, combined model, flag-all and flag-none strategies. The flag-all curve extends below the displayed vertical range at higher thresholds. Lower panels show combined-minus-structured net benefit with pointwise 95% patient-cluster bootstrap intervals conditional on fitted models. Thresholds were analyst specified and are not recommended clinical thresholds. Separate scales are used across endpoints; no intervention benefit was measured; Figure S2. Monthly documentation characteristics from April 2025 through June 2026. A: narrative character length among visits with text included in the primary cohort; the band is the interquartile range, not a confidence interval. B: missing narrative, pulse, systolic blood pressure and oxygen saturation among adult ESI 3–5 visits before exclusion for absent text. C: base-2 Jensen–Shannon divergence between monthly analyzed unigram/bigram distributions and the pooled development distribution, including one out-of-vocabulary bin. The dotted vertical line separates development from evaluation. These are descriptive changes; they do not identify their cause. Additional supporting files: TRIPOD + AI reporting map; Table S16 coefficient files; reproducibility archive containing executable analysis code and aggregate outputs. Patient-level records, original narratives and internal preparation notes are excluded.
