# Supplementary material for: Initial Emergency Department Triage Narratives for Predicting Hospital Admission and Early Critical Outcomes: A Retrospective Chronological Split Study
Source: J Clin Med. 2026 Oct 30;15(21):8477. doi: 10.3390/jcm15218477 (PMC13764153; doi:10.3390/jcm15218477)
Supplement: Supplementary file 1 [file jcm-15-08477-s001.zip › Supplementary Materials.pdf]

Supplementary Materials

Initial Emergency Department Triage Narratives for Predicting Hospital Admission and Early Critical Outcomes: A Retrospective Chronological-Split Study

This appendix provides the computational methods, cohort accounting and supplementary results for the retrospective chronological-split study. Development used visits from April–December 2025; evaluation used January–June 2026. The primary target is recorded admission. The main secondary target is general ICU transfer or index-hospital death within seven days of arrival. Tables S1–S16 and Figures S1–S2 document the reported analyses. S and T denote structured predictors and text, respectively; S+T denotes their combination. Detailed specifications support the concise Methods in the main manuscript.

Supplementary Methods

Source records and outcome boundaries

The exporting investigator confirmed selection of the initial completed TOPSOR/KSM assessment, including its narrative and measurements. The source system preserves that assessment when a separate retriage record is created. The local HIS release gate required completion before physician access. This account describes the implementation and export; the research data do not contain assessment identifiers, completion timestamps or an edit history, and no independent visit-level order/consultation/disposition chronology audit was performed. Later clinical fields were used only for outcome ascertainment or auditing.

Outcome linkage used the complete normalized visit-number/year key. Dated events were checked against ED arrival and available episode boundaries. The technical event tolerance was one minute before recorded arrival and two hours after an available hospital-end time; discrepant links were inspected computationally and excluded from the affected endpoints rather than repaired by an invented event time. Two evaluation visits had linked ICU records approximately 301–330 days before arrival and unresolved chronology. They were excluded from ICU and composite endpoints; they remained eligible for death-specific analyses because no death-linkage chronology conflict was identified. Direct ED-to-ICU transfers were included when documented in dated ward movements; event times were not imputed from ED departure. General ICU codes were A, OITP and OITS; the expanded sensitivity added OKW, a cardiac intensive-care and internal-medicine unit.

Movement and death-source coverage extended through 31 August 2026. A negative fixed-horizon label denotes no recorded index-hospital event within the specified interval; it does not verify survival after discharge or at another hospital. External transfers were excluded from hospital-outcome models. Completion of the hospital episode before 30 June was not required. The admission-duration sensitivity required an internal admission and at least 24 hours between ED departure and hospital discharge; external transfers and unresolved inpatient durations were excluded.

Preprocessing and fixed models

Symptoms and circumstances were joined with a space, lowercased, normalized by explicit ł-to-l replacement and Unicode NFKD decomposition with removal of combining marks, and reduced to single whitespace separators. No stemming, spelling correction, contextual language encoder or explicit negation model was used. Tokens matched (?u)\b\w\w+\b, so one-character words were omitted before formation of adjacent retained-token pairs. TF-IDF used word unigrams and bigrams, sublinear term frequency, smooth inverse document frequency, L2 document normalization, minimum document frequency 10, maximum document frequency 0.98 and at most 20,000 features. The vocabulary and all frequency weights were learned from development data only.

The fixed vectorizer stop list was: przyjecie, przyjecia, przyjety, przyjeta, przyjeto, oddzial, oddzialu, oddziale, skierowanie, celem. The following expressions were applied to normalized text. They are displayed in the same form as the executable source; workflow masking tests sensitivity to those expressions, not source chronology.

Standard admission and ward masking:

```
\b(?:przyjec\w* na oddzial\w*|do przyjecia|celem przyjecia|skierowan\w* do oddzial\w*|oddzial\w*)\b
```

Strict masking:

\b(?:przyjec\w*|hospitaliz\w*|oddzial\w*|oit\w*|icu\w*|wypis\w*|przekazan\w*|transfer\w*|konsultac\w*|skonsultow\w*|zakwalifik\w*|decyzj\w*|brak miejsc\w*|oczek\w* na miejsc\w*)\b

Additional pathway masking after strict masking:

\b(?:skierow\w*|kierowan\w*|kieruj\w*|poradn\w*|konsult\w*|ustalon\w*|umow\w*|planow\w*|kontrol\w*|operac\w*|zabieg\w*|ortoped\w*|okulist\w*|laryngol\w*|kardiolog\w*|chirurg\w*|neurochirurg\w*|urolog\w*|ginekolog\w*|hematolog\w*|onkolog\w*|psychiatr\w*|gastrolog\w*|neurolog\w*|dr|crp|rtg|tk|usg|mri)\b

The additional pathway expression was defined after inspection of admission coefficients and before its refit; evaluation scores did not select its terms. It includes referral, specialty, procedure and investigation vocabulary, including information that may legitimately be available at triage. It is an analyst-defined stress test. Two clinicians did not independently classify terms while blinded to coefficient sign, and no clinician-adjudicated category-removal analysis is claimed. The lexical representation was chosen as a transparent reference for estimating incremental information; superiority over contemporary Polish-language contextual encoders was not investigated.

Measured values outside the technical ranges in Table S5 were set to missing. Development-set median imputation retained all numeric features and added missingness flags. Numeric variables and flags were scaled using StandardScaler(with_mean=False). ESI used all three indicator columns (3, 4 and 5), with no dropped reference category. Logistic regression used L2 regularization, C=0.5, liblinear, intercept_scaling=100, max_iter=2000 and random_state=20260916. No class weighting or oversampling was used. All preprocessing was re-estimated inside each development cross-validation fold. Model coefficients, vocabulary and preprocessing were not fitted to 2026 outcomes. Prior project inspection of 2026 data still precludes an untouched confirmatory interpretation. The broad plausibility bounds screen gross technical errors rather than define clinical safety. Median replacement and missingness indicators were not used to claim recovery of unrecorded physiology under a missing-at-random assumption.

Performance and uncertainty

AUROC measures pairwise ranking; AP is average precision, the non-interpolated precision–recall summary. Brier score is mean squared probability error. Calibration intercept and slope jointly regress the observed outcome on the logit of predicted probability; ideal values are 0 and 1. Calibration-in-the-large fits an intercept with the predicted logit as an offset. O/E divides observed events by the sum of predicted risks. ECE is the sample-size-weighted absolute difference between observed and mean predicted risks in up to ten approximately equal-frequency probability groups, retaining prediction ties. Three-level ESI predictions therefore have only three distinct risk groups.

Paired patient-cluster bootstrapping used 500 resamples for main estimates and 300 for subgroups. Linked visits of the same patient were sampled together; records without a patient linkage became singleton clusters. Intervals are percentile 95% intervals conditional on the fitted models, not a bootstrap of the complete development procedure. AUROC and AP point estimates and intervals were suppressed below ten events or ten non-events. Five-fold stratified grouped cross-validation generated development out-of-fold predictions for target sensitivities of 80%, 90% and 95%. The selected thresholds were applied unchanged to evaluation; they are exploratory workload descriptors rather than recommended policies.

Net benefit was $TP/N - FP/N \times pt/(1-pt)$ at analyst-defined threshold probability pt. Assumed actions were earlier inpatient-bed coordination for admission risk and prioritized clinician reassessment for the seven-day composite. Threshold ranges were 1-30% and 0.1-2%, respectively; they were not clinically elicited. Models were compared with flag-all and flag-none strategies. Five hundred paired patient-cluster bootstrap resamples provided pointwise intervals conditional on fitted models. Net benefit represents an assumed tradeoff, not an observed clinical effect.

The offline runtime experiment used 25 warm-up calls followed by 500 sequential single-record calls and excluded database retrieval, network traffic, authentication, concurrent load and clinical queueing. Warmed admission prediction took a mean of 3.00 ms (95th percentile 3.79 ms); seven-day prediction took a mean of 2.90 ms (95th percentile 3.70 ms). These are in-memory preprocessing and prediction timings, not end-to-end HIS latency (Table S15). The analysis archive provides environment information, executed specifications and aggregate outputs; private predictions, source narratives and full model objects are not publicly distributed.

Additional analysis specifications

Five fixed explanatory predictor sets separated the contribution of measurement values and availability: core predictors (age, recorded sex, ESI and ambulance arrival); core plus seven measurement-missingness flags; core plus imputed measurements without explicit flags; core plus text; and core plus flags and text. Table S10 also reports the full structured and combined models. Imputed values can still encode missingness even when explicit flags are absent. Both main comparator models used the same text-evaluable cohort; visits lacking both narrative fields were described separately.

Narrative sensitivities refitted the models using strict masking, symptoms alone and the additional pathway lexicon. Documentation-period sensitivities refitted development after excluding July-August 2025 and, separately, using December 2025 only; both were evaluated on the same eligible 2026 visits. Other analyses excluded external transfers or non-routine dispositions, restricted evaluation to January-May, or used the first evaluation visit of each linkable patient absent from the eligible development cohort. Missing development identifiers limit the latter restriction. These variants were specified retrospectively and are reported together rather than used to choose a replacement model on evaluation performance (Table S9).

Monthly narrative length, out-of-vocabulary frequency and base-2 Jensen-Shannon divergence describe documentation change. The reference distribution used pooled development token frequencies in the development vocabulary plus one out-of-vocabulary bin. Out-of-vocabulary frequency includes terms excluded by development min_df or max_df and is not a linguistic-error rate. Divergence rose from 0.025 in November 2025 to 0.325 in December, while the out-of-vocabulary occurrence fraction rose from 10.7% to 25.0%. The cause of the July-August text gap and December measurement and narrative changes was not established (Table S13; Figure S2).

Apparent, grouped out-of-fold and chronological performance are compared in Table S12. Differences combine potential overfitting, smaller within-fold training samples and calendar changes; they are not a formal optimism-corrected external validation. The main paired and subgroup intervals condition on fitted development models and do not incorporate uncertainty from repeating the entire development procedure.

Supplementary Tables

Table S1. Cohort accounting and endpoint-specific denominators.

Stage or characteristic	Development	Evaluation
All source visits	64,789	47,526
Adults	62,618	45,495
Adults with ESI 3–5	57,140	40,507
No symptoms or circumstances text	5,405	74
Unknown disposition or ED departure among text-evaluable visits	0	0
Primary admission cohort	51,735	40,433
Recorded admissions	4,852	3,517
Internal admissions	4,468	3,276
Accepted external transfers	384	241
ED deaths	9	6
Non-routine departures	1,740	1,619
Missing hospital-end timestamp	75	47
Missing patient linkage	22,926	5

Stage or characteristic	Development	Evaluation
ICU chronology exclusions	0	2

Table S1 continued. Events over eligible visits.

Endpoint	Development events/n	Evaluation events/n
ICU within 24 h	35/51,351	27/40,190
ICU within 72 h	53/51,351	43/40,190
ICU within 7 days	63/51,351	63/40,190
Death within 7 days	118/51,351	68/40,192
Death within 30 days	238/51,351	156/40,192
ICU/death within 7 days	167/51,351	123/40,190
ICU including OKW/death within 7 days	372/51,351	286/40,190
Internal admission with stay ≥24 h	4,206/51,276	3,102/40,145

The seven-day composite includes overlap between ICU and death: 14 development and 8 evaluation visits had both events. The separate death cohort retains two evaluation visits excluded from ICU-based endpoints. All death endpoints refer to the index hospital.

Table S2. Time from ED arrival to recorded events.

Period	Event	Window	Events	Median (IQR), h	Range, h
Development	ICU	All observed	96	49.1 (10.0–193.4)	1.1–797.6
Development	ICU	≤24 h	35	7.3 (5.8–11.4)	1.1–21.2
Development	ICU	≤72 h	53	11.8 (6.8–32.8)	1.1–68.1
Development	ICU	≤168 h	63	15.0 (7.0–48.8)	1.1–166.5
Development	ICU	≤720 h	94	48.8 (9.7–188.2)	1.1–641.6
Development	Death	All observed	266	189.6 (65.3–438.3)	1.4–1955.2
Development	Death	≤24 h	20	11.4 (8.3–16.6)	1.4–22.8
Development	Death	≤72 h	71	39.1 (19.6–49.5)	1.4–70.7
Development	Death	≤168 h	118	56.7 (29.5–104.3)	1.4–163.0
Development	Death	≤720 h	238	174.0 (57.5–332.0)	1.4–711.4
Evaluation	ICU	All observed	78	47.0 (13.6–135.9)	1.1–865.3
Evaluation	ICU	≤24 h	27	9.4 (5.9–13.6)	1.1–22.7
Evaluation	ICU	≤72 h	43	18.5 (7.3–34.4)	1.1–65.0
Evaluation	ICU	≤168 h	63	34.1 (11.2–84.5)	1.1–167.2
Evaluation	ICU	≤720 h	76	45.2 (13.5–133.6)	1.1–712.4
Evaluation	Death	All observed	176	222.6 (96.3–439.9)	3.0–1940.0
Evaluation	Death	≤24 h	15	15.0 (12.1–18.3)	3.0–23.5
Evaluation	Death	≤72 h	35	35.7 (16.2–52.0)	3.0–71.2
Evaluation	Death	≤168 h	68	71.0 (35.7–109.2)	3.0–150.9
Evaluation	Death	≤720 h	156	198.0 (80.5–344.0)	3.0–718.4

All-observed summaries describe available events during source coverage, not a model endpoint with a fixed horizon. The corresponding counts may exceed seven- or 30-day counts. Exact cardiac-unit timing summaries are supplied in event_timing_r1.csv.

Table S3. Sensitivity to a hospital-discharge-date restriction.

Period	Old cutoff	Visits	Admission n (%)	Age median (IQR)	ESI 3	7-day events
Development	Excluded	75	75 (100.0%)	68.0 (58.5–73.0)	85.3%	8
Development	Retained	51,660	4,777 (9.2%)	46.0 (32.0–67.0)	43.5%	159
Evaluation	Excluded	497	222 (44.7%)	55.0 (38.0–73.0)	61.4%	19
Evaluation	Retained	39,936	3,295 (8.3%)	45.0 (31.0–66.0)	44.2%	104

The restriction required hospital discharge by 30 June 2026 00:00 and was applied to the primary cohort before excluding external transfers. It would exclude 497 evaluation visits, including 222 admissions (44.7%), versus 8.25% among 39,936 retained visits. Among the 495 excluded visits not externally transferred, 19 had the seven-day composite. The primary fixed-horizon analysis does not require discharge by June end.

Table S4. Monthly source availability among adult ESI 3–5 visits.

Month	Visits	Text missing	Link missing n	Pulse missing	SBP missing	SpO_2 missing
2025-04	6,033	0.7%	6,033	71.6%	61.3%	68.4%
2025-05	5,956	0.8%	5,956	73.2%	63.7%	71.6%
2025-06	6,462	0.3%	6,462	73.6%	62.4%	69.6%
2025-07	6,591	51.3%	6,561	86.9%	82.9%	86.5%
2025-08	6,494	28.4%	2,751	81.8%	74.8%	80.2%
2025-09	6,244	0.2%	147	72.8%	63.2%	70.4%
2025-10	6,645	0.3%	103	69.4%	60.0%	67.0%
2025-11	6,171	0.4%	100	66.2%	55.4%	63.0%
2025-12	6,544	0.2%	141	50.1%	50.2%	43.6%
2026-01	6,778	0.1%	6	49.1%	49.5%	41.9%
2026-02	5,970	0.2%	7	47.8%	48.0%	42.0%
2026-03	6,704	0.2%	4	52.3%	52.4%	45.7%
2026-04	6,604	0.2%	6	51.8%	52.0%	44.7%
2026-05	7,227	0.2%	13	55.3%	55.5%	48.6%
2026-06	7,224	0.2%	10	56.5%	56.7%	50.0%

Denominators include visits with no narrative, before exclusion from the primary model cohort. Monthly DBP, temperature, pain and respiratory-rate availability is provided in monthly_source_availability_r1.csv. The cause of the observed gaps and changes was not established from these exports.

Table S5. Technical cleaning ranges and measurement availability.

Measurement	Retained range	Development available	Evaluation available
Pulse	20–300 /min	16,141 (31.2%)	19,309 (47.8%)
SBP	40–300 mmHg	20,642 (39.9%)	19,231 (47.6%)
DBP	20–200 mmHg	20,071 (38.8%)	19,231 (47.6%)
SpO_2	30–100 %	17,748 (34.3%)	22,014 (54.4%)
Temperature	25–45 °C	6,445 (12.5%)	5,085 (12.6%)
Pain	0–10 score	6,229 (12.0%)	14,290 (35.3%)
Respiratory rate	4–80 /min	2,173 (4.2%)	2,411 (6.0%)

Availability is after range cleaning in the text-evaluable primary cohort (51,735 and 40,433 visits). These broad analyst-defined bounds identify gross errors, not normal physiology or safe clinical values. Detailed before/after-cleaning counts are supplied in vital_missingness_before_after_cleaning.csv.

Table S6. Missing measurements by recorded admission.

Measurement	Dev non-admitted	Dev admitted	Eval non-admitted	Eval admitted
Pulse	70.3%	54.4%	54.5%	28.1%
SBP	61.9%	42.7%	54.7%	28.6%
DBP	63.0%	44.1%	54.7%	28.6%
SpO_2	67.1%	52.0%	47.4%	26.4%
Temperature	88.1%	81.9%	87.6%	85.3%
Pain	88.1%	86.3%	64.4%	67.6%
Respiratory rate	96.0%	93.9%	94.3%	91.4%

Percentages use the respective outcome-stratum denominator. Differential missingness does not by itself establish when a measurement was recorded.

Table S6 continued. Visits with and without narrative text.

Period	Text	Visits	Median age	ESI 3	Admitted	Pulse missing
Development	Absent	5,405	48.0	44.6%	9.3%	99.8%
Development	Present	51,735	46.0	43.6%	9.4%	68.8%
Evaluation	Absent	74	44.5	43.2%	13.5%	91.9%
Evaluation	Present	40,433	46.0	44.4%	8.7%	52.2%

Both main comparator models use the same text-evaluable cohort. Visits without text are described here rather than imputed as narratives.

Table S7. Full endpoint performance and calibration.

Endpoint	Model	AUROC (95% CI)	AP (95% CI)	Brier (95% CI)
Admission	ESI	0.681 (0.674–0.689)	0.138 (0.133–0.144)	0.07619 (0.07404–0.07859)
Admission	S	0.763 (0.755–0.771)	0.246 (0.234–0.259)	0.07242 (0.07032–0.07465)
Admission	T	0.800 (0.793–0.807)	0.292 (0.278–0.309)	0.07005 (0.06796–0.07224)
Admission	S+T	0.835 (0.828–0.841)	0.340 (0.325–0.357)	0.06696 (0.06497–0.06898)
ICU/death within 7 days	ESI	0.764 (0.746–0.777)	0.007 (0.005–0.008)	0.00304 (0.00248–0.00356)
ICU/death within 7 days	S	0.904 (0.884–0.923)	0.042 (0.026–0.083)	0.00302 (0.00248–0.00353)
ICU/death within 7 days	T	0.858 (0.828–0.887)	0.034 (0.020–0.066)	0.00303 (0.00247–0.00355)
ICU/death within 7 days	S+T	0.913 (0.897–0.930)	0.055 (0.031–0.096)	0.00300 (0.00247–0.00350)
ICU within 72 hours	S	0.846 (0.782–0.901)	0.012 (0.004–0.056)	0.00107 (0.00078–0.00137)

Endpoint	Model	AUROC (95% CI)	AP (95% CI)	Brier (95% CI)
ICU within 72 hours	S+T	0.851 (0.793–0.903)	0.012 (0.005–0.056)	0.00108 (0.00078–0.00137)
Death within 7 days	S	0.941 (0.926–0.956)	0.037 (0.021–0.085)	0.00169 (0.00130–0.00211)
Death within 7 days	S+T	0.948 (0.934–0.961)	0.045 (0.027–0.089)	0.00168 (0.00129–0.00209)
Death within 30 days	S	0.917 (0.895–0.938)	0.053 (0.039–0.080)	0.00380 (0.00326–0.00439)
Death within 30 days	S+T	0.930 (0.911–0.948)	0.069 (0.050–0.098)	0.00377 (0.00323–0.00435)
ICU including OKW/death within 7 days	S	0.877 (0.861–0.891)	0.048 (0.037–0.067)	0.00694 (0.00611–0.00768)
ICU including OKW/death within 7 days	S+T	0.899 (0.883–0.911)	0.061 (0.048–0.081)	0.00687 (0.00604–0.00762)
Internal admission with stay ≥24 h	S	0.774 (0.766–0.783)	0.232 (0.219–0.247)	0.06505 (0.06301–0.06703)
Internal admission with stay ≥24 h	S+T	0.842 (0.836–0.848)	0.321 (0.306–0.339)	0.06052 (0.05855–0.06240)

Intervals use paired patient-cluster bootstrap resampling conditional on fitted models. General ICU is used unless OKW is explicitly included. S = structured; T = text.

Table S7 continued. Probability calibration.

Endpoint	Model	Intercept	Slope	CITL	O/E (95% CI)	ECE
Admission	ESI	-0.045	1.027	-0.100	0.917 (0.886–0.949)	0.00856
Admission	S	0.019	1.036	-0.052	0.958 (0.928–0.989)	0.00429
Admission	T	0.365	1.186	-0.001	1.000 (0.967–1.034)	0.01216
Admission	S+T	0.125	1.083	-0.023	0.982 (0.954–1.013)	0.00621
ICU/death within 7 days	ESI	1.722	1.352	-0.079	0.924 (0.750–1.084)	0.00025
ICU/death within 7 days	S	-0.639	0.849	0.020	1.019 (0.822–1.202)	0.00066
ICU/death within 7 days	T	7.452	2.384	-0.075	0.928 (0.754–1.086)	0.00258
ICU/death within 7 days	S+T	-0.522	0.874	0.015	1.014 (0.817–1.196)	0.00066
ICU within 72 hours	S	-0.804	0.868	-0.047	0.955 (0.690–1.218)	0.00033
ICU within 72 hours	S+T	-0.786	0.870	-0.037	0.964 (0.697–1.228)	0.00026
Death within 7 days	S	-0.603	0.907	-0.203	0.825 (0.628–1.022)	0.00046
Death within 7 days	S+T	-0.449	0.946	-0.220	0.812 (0.621–1.005)	0.00039
Death within 30 days	S	-0.525	0.875	-0.040	0.963 (0.825–1.117)	0.00067
Death within 30 days	S+T	-0.328	0.927	-0.056	0.949 (0.815–1.104)	0.00047

Endpoint	Model	Intercept	Slope	CITL	O/E (95% CI)	ECE
ICU including OKW/death within 7 days	S	-0.385	0.908	-0.036	0.966 (0.845–1.070)	0.00138
ICU including OKW/death within 7 days	S+T	-0.091	0.980	-0.016	0.985 (0.860–1.094)	0.00076
Internal admission with stay ≥24 h	S	0.038	1.029	-0.021	0.982 (0.948–1.017)	0.00316
Internal admission with stay ≥24 h	S+T	0.173	1.082	0.015	1.012 (0.979–1.045)	0.00682

CITL = calibration-in-the-large. Ideal calibration intercept/CITL = 0; slope and O/E = 1. ECE uses up to ten probability groups with ties preserved. Endpoint-specific CSV files also report ECE and Brier confidence intervals and calibration-bin counts.

Table S8. Admission performance within clinical groups.

Group	Model	Visits	Events	AUROC (95% CI)	AP (95% CI)
ESI 3	S	17,972	2,719	0.685 (0.676–0.697)	0.281 (0.268–0.297)
ESI 3	S+T	17,972	2,719	0.762 (0.753–0.774)	0.372 (0.356–0.392)
ESI 4	S	18,220	658	0.671 (0.649–0.691)	0.074 (0.065–0.085)
ESI 4	S+T	18,220	658	0.822 (0.804–0.838)	0.200 (0.176–0.230)
ESI 5	S	4,241	140	0.675 (0.634–0.717)	0.093 (0.062–0.131)
ESI 5	S+T	4,241	140	0.884 (0.852–0.916)	0.340 (0.272–0.437)
Age 18–64	S	29,439	1,745	0.734 (0.721–0.746)	0.163 (0.150–0.177)
Age 18–64	S+T	29,439	1,745	0.831 (0.822–0.839)	0.271 (0.254–0.293)
Age 65–74	S	5,308	714	0.728 (0.707–0.749)	0.302 (0.273–0.332)
Age 65–74	S+T	5,308	714	0.805 (0.789–0.822)	0.396 (0.363–0.436)
Age 75–84	S	3,962	683	0.700 (0.680–0.720)	0.316 (0.285–0.352)
Age 75–84	S+T	3,962	683	0.770 (0.754–0.789)	0.404 (0.369–0.443)
Age ≥85	S	1,724	375	0.659 (0.621–0.688)	0.332 (0.292–0.382)
Age ≥85	S+T	1,724	375	0.727 (0.696–0.753)	0.410 (0.366–0.464)
Female	S	20,883	1,693	0.748 (0.735–0.760)	0.222 (0.208–0.241)
Female	S+T	20,883	1,693	0.830 (0.819–0.840)	0.330 (0.309–0.354)
Male	S	19,550	1,824	0.777 (0.766–0.787)	0.268 (0.249–0.288)
Male	S+T	19,550	1,824	0.838 (0.829–0.847)	0.350 (0.327–0.373)

Discrimination point estimates and intervals are suppressed with fewer than ten events or non-events. Internally admitted-patient results condition on eventual disposition. Subgroup files also report calibration summaries; these should be interpreted cautiously with small event counts.

Table S8 continued. ICU/death within 7 days performance within clinical groups.

Group	Model	Visits	Events	AUROC (95% CI)	AP (95% CI)
ESI 3	S	17,808	119	0.813 (0.777–0.848)	0.047 (0.027–0.088)
ESI 3	S+T	17,808	119	0.828 (0.795–0.861)	0.058 (0.032–0.104)
ESI 4	S	18,160	3	Suppressed	Suppressed
ESI 4	S+T	18,160	3	Suppressed	Suppressed
ESI 5	S	4,222	1	Suppressed	Suppressed
ESI 5	S+T	4,222	1	Suppressed	Suppressed
Age 18–64	S	29,274	33	0.922 (0.889–0.953)	0.020 (0.009–0.049)
Age 18–64	S+T	29,274	33	0.936 (0.910–0.960)	0.025 (0.012–0.056)
Age 65–74	S	5,276	26	0.824 (0.738–0.903)	0.063 (0.016–0.158)
Age 65–74	S+T	5,276	26	0.841 (0.765–0.911)	0.070 (0.018–0.169)
Age 75–84	S	3,923	40	0.773 (0.696–0.829)	0.074 (0.022–0.179)
Age 75–84	S+T	3,923	40	0.781 (0.711–0.837)	0.086 (0.024–0.187)
Age ≥85	S	1,717	24	0.787 (0.698–0.859)	0.050 (0.025–0.098)
Age ≥85	S+T	1,717	24	0.805 (0.723–0.877)	0.068 (0.032–0.148)
Female	S	20,757	56	0.890 (0.851–0.920)	0.028 (0.016–0.058)
Female	S+T	20,757	56	0.901 (0.867–0.927)	0.032 (0.019–0.063)
Male	S	19,433	67	0.915 (0.888–0.938)	0.075 (0.031–0.131)
Male	S+T	19,433	67	0.924 (0.901–0.944)	0.085 (0.037–0.144)
Internally admitted	S	3,276	117	0.742 (0.704–0.780)	0.112 (0.079–0.164)
Internally admitted	S+T	3,276	117	0.750 (0.715–0.788)	0.116 (0.080–0.168)

Discrimination point estimates and intervals are suppressed with fewer than ten events or non-events. Internally admitted-patient results condition on eventual disposition. Subgroup files also report calibration summaries; these should be interpreted cautiously with small event counts.

Complete component-endpoint subgroup results, including ESI, age, sex and internally admitted strata where applicable, are provided in icu72_subgroups.csv, death7_subgroups.csv and death30_subgroups.csv. Expanded-unit and duration sensitivities have corresponding subgroup files. The same suppression rule applies in these files; a blank discrimination value is intentional, not a zero estimate.

Table S9. Admission sensitivity analyses.

Analysis	Model	Dev visits	Eval visits	AUROC	AP
Strict masking	S+T	51,735	40,433	0.836	0.345
Symptoms only	S+T	51,735	40,433	0.836	0.344
Development excluding Jul–Aug	S	43,871	40,433	0.763	0.246
Development excluding Jul–Aug	S+T	43,871	40,433	0.832	0.333

Analysis	Model	Dev visits	Eval visits	AUROC	AP
December-only development	S	6,530	40,433	0.759	0.244
December-only development	S+T	6,530	40,433	0.801	0.279
Routine dispositions, excluding ED deaths	S	51,735	38,808	0.761	0.251
Routine dispositions, excluding ED deaths	S+T	51,735	38,808	0.834	0.347
No external transfers	S	51,735	40,192	0.771	0.241
No external transfers	S+T	51,735	40,192	0.841	0.335
New linked patients, first visit	S	51,735	29,794	0.770	0.245
New linked patients, first visit	S+T	51,735	29,794	0.837	0.335
January–May evaluation	S	51,735	33,220	0.760	0.245
January–May evaluation	S+T	51,735	33,220	0.832	0.341

All specified variants are reported. Refit variants estimate parameters using the designated development data only. Evaluation restrictions apply the fitted primary models unless a distinct endpoint is being modelled. Exact event counts and calibration are in the corresponding sensitivity CSV.

Table S9 continued. ICU/death within 7 days sensitivity analyses.

Analysis	Model	Dev visits	Eval visits	AUROC	AP
Strict masking	S+T	51,351	40,190	0.913	0.056
Symptoms only	S+T	51,351	40,190	0.913	0.055
Development excluding Jul–Aug	S	43,544	40,190	0.906	0.041
Development excluding Jul–Aug	S+T	43,544	40,190	0.915	0.053
December-only development	S	6,466	40,190	0.867	0.037
December-only development	S+T	6,466	40,190	0.872	0.040
Routine dispositions, excluding ED deaths	S	51,351	38,565	0.902	0.042
Routine dispositions, excluding ED deaths	S+T	51,351	38,565	0.911	0.055
No external transfers	S	51,351	40,190	0.904	0.042
No external transfers	S+T	51,351	40,190	0.913	0.055
New linked patients, first visit	S	51,351	29,654	0.917	0.046
New linked patients, first visit	S+T	51,351	29,654	0.927	0.060
January–May evaluation	S	51,351	33,018	0.906	0.040
January–May evaluation	S+T	51,351	33,018	0.914	0.054
Earlier completion restriction	S	51,351	39,695	0.916	0.042
Earlier completion restriction	S+T	51,351	39,695	0.924	0.058

All specified variants are reported. Refit variants estimate parameters using the designated development data only. Evaluation restrictions apply the fitted primary models unless a distinct endpoint is being modelled. Exact event counts and calibration are in the corresponding sensitivity CSV.

Table S9 continued. Expanded pathway masking and alternative endpoint definitions.

Endpoint	Model or variant	Visits	Events	AUROC (95% CI)	AP (95% CI)
Admission	Pathway vocabulary removed	40,433	3,517	0.835 (0.829–0.842)	0.348 (0.333–0.364)
ICU/death within 7 days	Pathway vocabulary removed	40,190	123	0.914 (0.897–0.930)	0.055 (0.031–0.096)
ICU including OKW/death within 7 days	S	40,190	286	0.877 (0.861–0.891)	0.048 (0.037–0.067)
ICU including OKW/death within 7 days	S+T	40,190	286	0.899 (0.883–0.911)	0.061 (0.048–0.081)
Internal admission with stay ≥24 h	S	40,145	3,102	0.774 (0.766–0.783)	0.232 (0.219–0.247)
Internal admission with stay ≥24 h	S+T	40,145	3,102	0.842 (0.836–0.848)	0.321 (0.306–0.339)

Pathway masking is analyst-defined and was not based on two blinded clinician classifications. The additional lexicon changed 9,413 development and 3,040 evaluation admission narratives; 11 and 6 became empty after masking, respectively, but remained in the sensitivity cohort. Cardiac-unit inclusion expands the available ward definition. Inpatient stay ≥24 hours remains an operational proxy rather than adjudicated medical need.

Table S10. Fixed predictor-set and missingness comparisons.

Endpoint	Predictor set	AUROC (95% CI)	AP (95% CI)	Brier
Admission	Core	0.752 (0.743–0.760)	0.222 (0.210–0.235)	0.07347
Admission	Core + flags	0.754 (0.745–0.762)	0.224 (0.212–0.237)	0.07336
Admission	Core + values	0.762 (0.753–0.770)	0.245 (0.233–0.258)	0.07252
Admission	Core + text	0.832 (0.825–0.838)	0.332 (0.317–0.349)	0.06743
Admission	Core + flags + text	0.832 (0.825–0.838)	0.333 (0.317–0.349)	0.06741
Admission	S	0.763	0.246	0.07242
Admission	S+T	0.835	0.340	0.06696
ICU/death within 7 days	Core	0.894 (0.873–0.912)	0.022 (0.016–0.031)	0.00302
ICU/death within 7 days	Core + flags	0.891 (0.869–0.909)	0.021 (0.015–0.031)	0.00302
ICU/death within 7 days	Core + values	0.908 (0.890–0.925)	0.044 (0.026–0.083)	0.00302
ICU/death within 7 days	Core + text	0.912 (0.894–0.927)	0.039 (0.025–0.067)	0.00300
ICU/death within 7 days	Core + flags + text	0.909 (0.890–0.925)	0.035 (0.023–0.057)	0.00300
ICU/death within 7 days	S	0.904	0.042	0.00302
ICU/death within 7 days	S+T	0.913	0.055	0.00300

Core = age, sex, ambulance arrival and ESI. Flags = seven measurement-missingness indicators. Values = imputed measured values without explicit indicators. S = core, values and flags; S+T adds text. Core+text omits measured values and flags. Exact paired differences and calibration are supplied in *_missingness_ablation_differences.csv and *_missingness_ablation.csv.

Table S11. Admission operating points.

Model	Dev target	Risk threshold	Sensitivity	Specificity	PPV	NPV	Flags/1000
S	80%	6.415%	80.9%	56.7%	15.12%	96.89%	465.5
S	90%	4.185%	90.2%	40.1%	12.54%	97.72%	625.6
S	95%	3.131%	94.7%	27.0%	11.00%	98.15%	748.8
S+T	80%	8.615%	78.4%	72.9%	21.61%	97.26%	315.8
S+T	90%	5.422%	88.8%	59.4%	17.25%	98.24%	447.7
S+T	95%	3.733%	93.9%	47.5%	14.56%	98.79%	560.8

Table S11 continued. ICU/death within 7 days operating points.

Model	Dev target	Risk threshold	Sensitivity	Specificity	PPV	NPV	Flags/1000
S	80%	0.487%	72.4%	88.9%	1.96%	99.90%	112.8
S	90%	0.199%	87.0%	79.8%	1.30%	99.95%	204.3
S	95%	0.099%	93.5%	68.1%	0.89%	99.97%	320.8
S+T	80%	0.530%	70.7%	89.8%	2.09%	99.90%	103.5
S+T	90%	0.194%	88.6%	79.8%	1.33%	99.96%	204.5
S+T	95%	0.103%	93.5%	69.5%	0.93%	99.97%	307.3

Table S11 continued. Confusion counts and false-positive burden.

Endpoint	Model	Dev target	TP	FP	FN	TN	FP/TP
Admission	S	80%	2,845	15,977	672	20,939	5.6
Admission	S	90%	3,172	22,123	345	14,793	7.0
Admission	S	95%	3,329	26,947	188	9,969	8.1
Admission	S+T	80%	2,759	10,009	758	26,907	3.6
Admission	S+T	90%	3,123	14,977	394	21,939	4.8
Admission	S+T	95%	3,302	19,374	215	17,542	5.9
ICU/death within 7 days	S	80%	89	4,443	34	35,624	49.9
ICU/death within 7 days	S	90%	107	8,102	16	31,965	75.7
ICU/death within 7 days	S	95%	115	12,779	8	27,288	111.1
ICU/death within 7 days	S+T	80%	87	4,072	36	35,995	46.8
ICU/death within 7 days	S+T	90%	109	8,108	14	31,959	74.4
ICU/death within 7 days	S+T	95%	115	12,234	8	27,833	106.4

Thresholds were chosen from development out-of-fold predictions to meet the stated target, then applied unchanged to evaluation. Actual evaluation sensitivity can differ. FN denotes missed endpoint events. These are exploratory operating points, not clinical recommendations.

Table S12. Apparent, grouped out-of-fold and chronological performance.

Endpoint	Model	Prediction set	AUROC	AP	Brier
Admission	S	Apparent development	0.754	0.244	0.07794
Admission	S	Grouped out-of-fold	0.753	0.241	0.07810
Admission	S	Chronological evaluation	0.763	0.246	0.07242
Admission	S+T	Apparent development	0.868	0.434	0.06694
Admission	S+T	Grouped out-of-fold	0.826	0.335	0.07241
Admission	S+T	Chronological evaluation	0.835	0.340	0.06696
ICU/death within 7 days	S	Apparent development	0.923	0.088	0.00310
ICU/death within 7 days	S	Grouped out-of-fold	0.913	0.072	0.00313
ICU/death within 7 days	S	Chronological evaluation	0.904	0.042	0.00302
ICU/death within 7 days	S+T	Apparent development	0.947	0.157	0.00298
ICU/death within 7 days	S+T	Grouped out-of-fold	0.918	0.087	0.00311
ICU/death within 7 days	S+T	Chronological evaluation	0.913	0.055	0.00300

Five-fold development cross-validation refitted all preprocessing. Differences combine overfitting, smaller within-fold training sets and calendar changes; this table is not a formal optimism-corrected external validation. Incomplete development linkage limits patient grouping.

Table S13. Monthly narrative length and vocabulary drift.

Month	Visits	Characters median (IQR)	Words median (IQR)	Outside vocabulary	JS divergence
2025-04	5,989	109 (53–188)	17 (8–30)	13.15%	0.014
2025-05	5,909	101 (54–188)	16 (9–29)	13.69%	0.017
2025-06	6,441	100 (49–191)	16 (8–30)	13.19%	0.015
2025-07	3,213	155 (68–206)	25 (11–32)	11.07%	0.025
2025-08	4,651	106 (51–194)	17 (8–30)	12.94%	0.016
2025-09	6,229	131 (53–198)	20 (8–31)	11.58%	0.016
2025-10	6,628	153 (55–204)	24 (9–32)	10.05%	0.018
2025-11	6,145	124 (52–211)	19 (8–33)	10.73%	0.025
2025-12	6,530	55 (35–83)	9 (6–13)	24.97%	0.325

Month	Visits	Characters median (IQR)	Words median (IQR)	Outside vocabulary	JS divergence
2026-01	6,769	54 (34–82)	8 (5–12)	24.80%	0.334
2026-02	5,958	53 (33–79)	8 (5–12)	25.45%	0.336
2026-03	6,692	50 (31–76)	8 (5–12)	25.15%	0.335
2026-04	6,592	50 (31–74)	8 (5–11)	24.80%	0.338
2026-05	7,209	47 (29–73)	7 (5–11)	24.46%	0.338
2026-06	7,213	47 (29–72)	7 (5–11)	25.42%	0.337

These summaries concern the text-evaluable primary cohort. "Words" are whitespace-separated units; the model analyzer uses its documented token and bigram rules. JS is base-2 Jensen–Shannon divergence against pooled development frequencies including one outside-vocabulary bin. An unchanged record-locking mechanism does not imply unchanged documentation completeness.

Table S14. Admission conditional decision analysis.

Risk threshold	S net benefit/1000	S+T net benefit/1000	Difference/1000 (95% CI)
1.0%	77.76	78.38	0.615 (0.461–0.755)
2.0%	68.66	71.82	3.158 (2.684–3.583)
3.0%	61.58	66.30	4.717 (4.132–5.413)
5.0%	50.00	57.79	7.791 (6.815–8.840)
7.5%	38.85	48.99	10.139 (8.895–11.419)
10.0%	30.32	41.02	10.704 (9.287–12.042)
15.0%	19.33	29.43	10.097 (8.587–11.505)
20.0%	11.21	21.61	10.400 (8.789–12.090)
25.0%	6.13	15.47	9.341 (7.575–10.873)
30.0%	2.52	10.25	7.731 (6.094–9.293)

Pointwise patient-cluster bootstrap intervals condition on fitted models. The assumed threshold/action tradeoff was analyst-defined. Net benefit is not a measured intervention effect. All strategy-level estimates and intervals are provided in *_decision_curves.csv.

Table S14 continued. ICU/death within 7 days conditional decision analysis.

Risk threshold	S net benefit/1000	S+T net benefit/1000	Difference/1000 (95% CI)
0.10%	2.55	2.55	0.004 (0.003–0.006)
0.20%	2.26	2.29	0.031 (-0.044–0.108)
0.30%	2.02	2.11	0.084 (0.009–0.181)
0.50%	1.64	1.64	-0.008 (-0.159–0.119)
0.75%	1.22	1.29	0.070 (0.016–0.157)
1.00%	1.05	1.07	0.019 (-0.152–0.164)
1.50%	0.65	0.84	0.182 (0.038–0.353)

Risk threshold	S net benefit/1000	S+T net benefit/1000	Difference/1000 (95% CI)
2.00%	0.36	0.48	0.122 (-0.005–0.257)

Pointwise patient-cluster bootstrap intervals condition on fitted models. The assumed threshold/action tradeoff was analyst-defined. Net benefit is not a measured intervention effect. All strategy-level estimates and intervals are provided in *_decision_curves.csv.

Table S15. Offline single-record prediction runtime.

Endpoint	Model	Calls	Mean ms	Median ms	P95 ms	Maximum ms
Admission	S	500	2.126	2.029	2.634	6.883
Admission	S+T	500	2.997	2.788	3.786	11.486
ICU/death within 7 days	S	500	2.068	1.992	2.520	7.244
ICU/death within 7 days	S+T	500	2.902	2.738	3.704	5.888

Twenty-five warm-up calls preceded 500 timed calls per model. Timing includes in-memory fitted preprocessing and prediction only. It excludes database/network access, authentication, concurrency and clinical queueing; it is not production-system latency.

Table S16. Coefficient files provided with the supplement.

Endpoint	Filename stem	Text rows	Structured rows
Admission	admission	100	21
ICU/death within 7 days	critical7	100	21
ICU within 72 hours	icu72	100	21
Death within 7 days	death7	100	21
Death within 30 days	death30	100	21
ICU including OKW/death within 7 days	critical7_cardiac	100	21
Internal admission with stay ≥24 h	admission24h	100	21

Files are in Table_S16_Coefficients/. For each listed stem, the text file is <stem>_top100_text_terms.csv, the structured file is <stem>_structured_coefficients.csv, and preprocessing metadata are in <stem>_numeric_imputation_and_scaling.csv. Each text file contains 50 positive and 50 negative terms with development document frequencies. Structured files contain the coefficients from the combined model, scaling information and its intercept; “structured rows” includes that intercept.

Text coefficients multiply the L2-normalized TF-IDF inputs. Document frequency counts development records containing the term after the specified preprocessing. The selected list is not the full learned vocabulary. One geographic token is obscured in public coefficient files to minimize identifying vocabulary; its coefficient and document frequency are retained, and the fitted models were not changed. Numeric-pipeline coefficients apply to scaled values or missingness flags; the supplied original-unit coefficient divides by the fitted scale. All three ESI indicators are retained, so their coefficients are not contrasts against a dropped ESI reference level. The constant model intercept is reported separately. The seven endpoint-specific numeric preprocessing files specify development imputation medians, source fields, missingness indicators, fitted scales and lack of centering; the combined all_numeric_imputation_and_scaling.csv contains 119 rows. Correlated predictors, scaling and ridge penalization limit comparisons based on coefficient magnitude alone. No independent blinded two-clinician classification or agreement statistic is reported.

The intended analysis archive contains code, executed analytical specifications and aggregate results. Internal preparation notes, draft correspondence, patient-level records, source narratives and private model objects are excluded. The manuscript response to reviewers is supplied separately.

Supplementary Figures

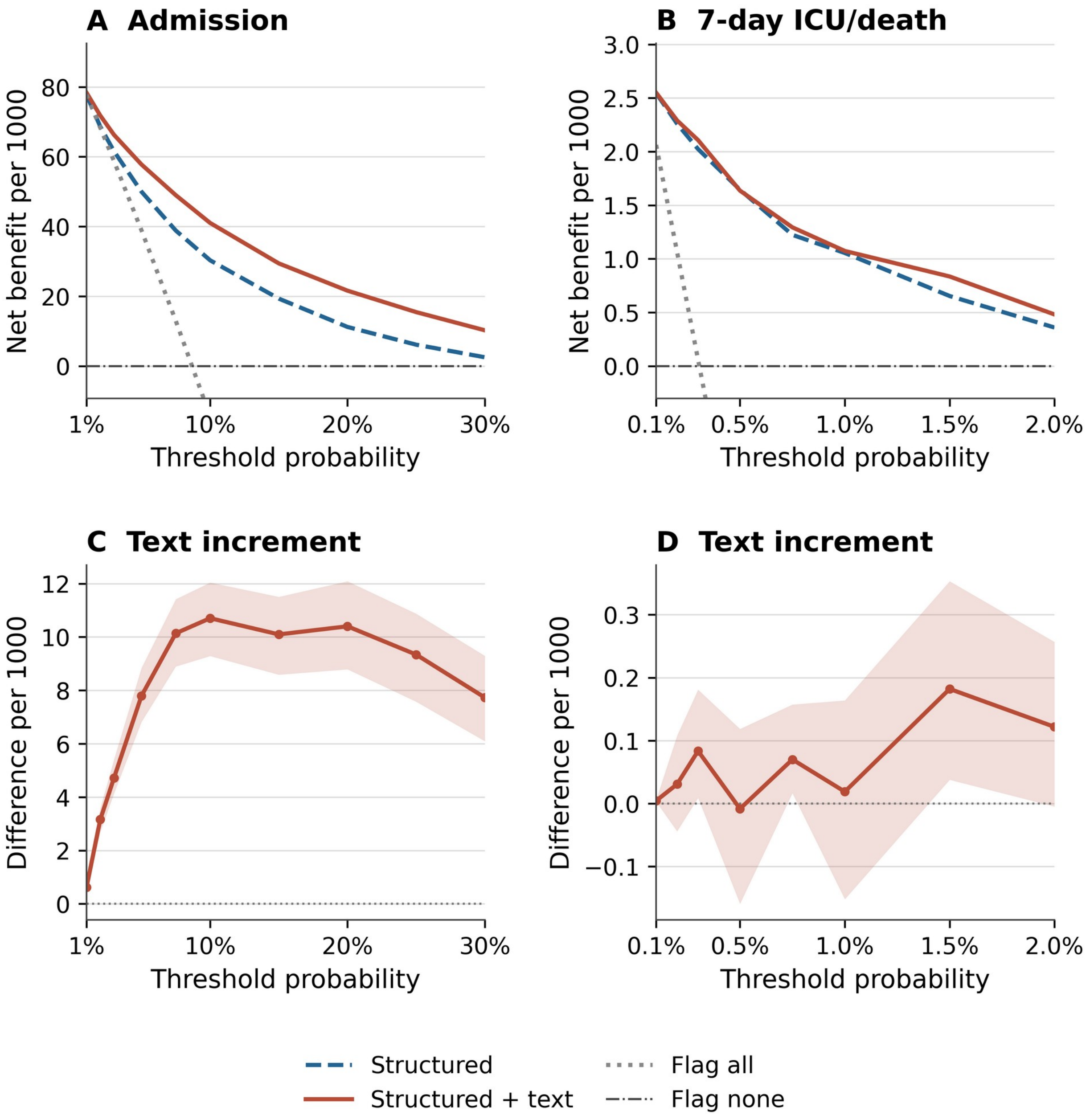

Figure S1. Exploratory decision curves for hypothetical actions. Top panels show net benefit per 1000 visits for the structured model, combined model, flag-all and flag-none strategies. The flag-all curve extends below the displayed vertical range at higher thresholds. Lower panels show combined-minus-structured net benefit with pointwise 95% patient-cluster bootstrap intervals conditional on fitted models. Thresholds were analyst specified and are not recommended clinical thresholds. Separate scales are used across endpoints; no intervention benefit was measured.

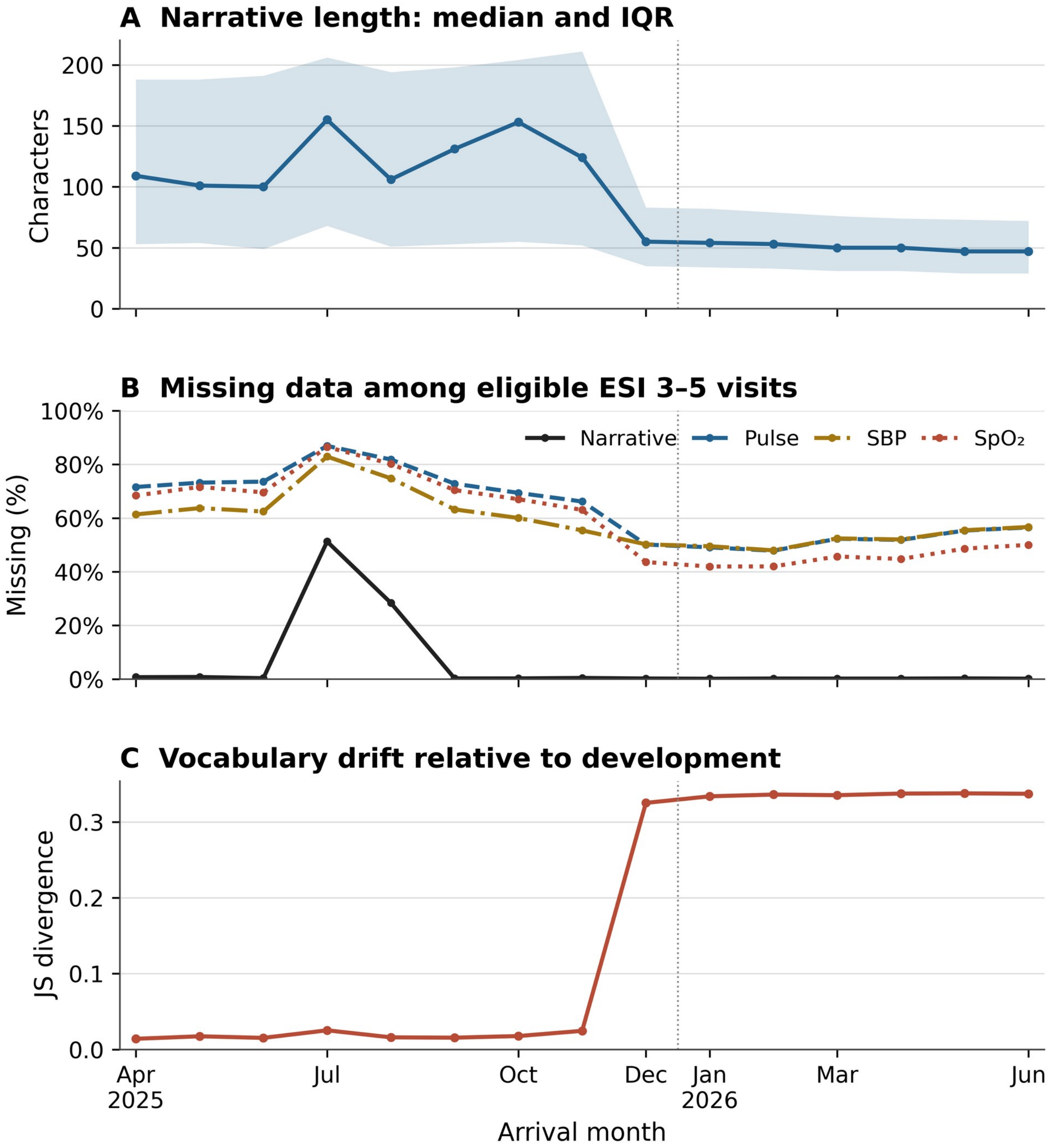

Figure S2. Monthly documentation characteristics from April 2025 through June 2026. A: narrative character length among visits with text included in the primary cohort; the band is the interquartile range, not a confidence interval. B: missing narrative, pulse, systolic blood pressure and oxygen saturation among adult ESI 3–5 visits before exclusion for absent text. C: base-2 Jensen–Shannon divergence between monthly analyzed unigram/bigram distributions and the pooled development distribution, including one out-of-vocabulary bin. The dotted vertical line separates development from evaluation. These are descriptive changes; they do not identify their cause.
