# Supplementary material for: Initial Emergency Department Triage Narratives for Predicting Hospital Admission and Early Critical Outcomes: A Retrospective Chronological Split Study
Source: J Clin Med. 2026 Oct 30;15(21):8477. doi: 10.3390/jcm15218477 (PMC13764153; doi:10.3390/jcm15218477)
Supplement: Supplementary file 1 [file jcm-15-08477-s001.zip › TRIPOD_AI_Reporting_Map_R2.pdf]

TRIPOD AI Reporting Map

Initial emergency department triage narratives for admission and early critical outcomes

This map covers the 52 main items and 13 abstract items of TRIPOD+AI. It identifies reported information and remaining evidence gaps in the revised retrospective chronological-split study. It is not a claim of full compliance or a risk-of-bias score. Brief requirement cues are paraphrases; the official checklist provides the complete wording.

Reported means that information is described within the study scope; it does not remove a design limitation. Partial and incomplete entries identify missing information or evidence. Not applicable denotes a procedure that was not undertaken.

Section labels refer to the main manuscript unless a supplementary table or archive is specified. Source record locking, chronological evaluation and reporting completeness should not be interpreted as independent verification of every record or as external validation.

Title abstract and rationale

Item and cue	Location	Status	Evidence and remaining limits
1 Model, population and outcome	Title; Abstract	Partial	The title identifies ED triage narratives, admission, early critical outcomes and retrospective chronological splitting. Adult and ESI 3–5 restrictions are explicit in the abstract but not in the title.
2 Informative abstract	Abstract; abstract-specific map below	Partial	The structured abstract identifies the retrospective setting, prior data inspection, chronological periods, available-text cohort, penalized logistic comparison, admission and seven-day outcomes, main results and critical-endpoint workload. Some predictor details, secondary confidence intervals and the secondary development denominator are omitted; see the abstract map.
3a Clinical rationale	Introduction	Reported	The introduction explains incremental information from initial Polish triage narratives, cites combined structured/text prediction studies, and distinguishes temporal eligibility from workflow-related label information.
3b Purpose and intended users	Introduction; Methods 2.7; Discussion 4.1-4.2; Supplementary Methods	Reported for hypothetical use	Recorded admission is a local disposition decision. Earlier bed coordination and prioritized clinician reassessment are assumed future actions; neither workflow nor decision impact was tested.
3c Health inequalities	Methods 2.6; Discussion 4.3	Partial	Age, recorded sex and ESI subgroup analyses are reported within the available data. No comprehensive fairness assessment, additional sociodemographic analysis or effect on care inequalities was performed.
4 Study objectives	Introduction; Methods 2.1 and 2.3	Reported	Primary recorded admission and secondary early hospital outcomes are distinguished. The study assesses incremental text information across a retrospective chronological split, without claiming independent confirmatory validation.

Data sources participants and measurements

Item and cue	Location	Status	Evidence and remaining limits
5a Sources and representation	Methods 2.1–2.3; Supplementary source and cohort accounting; Discussion 4.3	Reported with limitations	Routine single-hospital triage, disposition, ward-movement and death sources are described. Adult ESI 3–5 and nonempty-text eligibility limit representation of all ED visits; upstream first-assessment selection is exporter-confirmed.
5b Accrual and outcome coverage	Methods 2.1 and 2.3; Supplementary source-coverage audit	Reported	Arrivals span 1 April 2025–30 June 2026. Development is April–December 2025 and evaluation January–June 2026. Hospital-event sources extend through 31 August 2026, beyond the longest 30-day horizon; post-discharge and outside-hospital outcomes remain unobserved.
6a Care setting	Methods 2.1	Reported	One university-hospital ED in Poznań, Poland, is identified. No prehospital or second-hospital validation was conducted.
6b Eligibility	Methods 2.1 and 2.3; Figure 1; Supplementary cohort accounting	Reported	Adult ESI 3–5 visits require an initial narrative, recorded disposition and ED departure. Hospital-outcome analyses exclude external transfers, with endpoint-specific eligibility. Completion of the entire hospital episode before June end is not required. Two evaluation visits with unresolved ICU-event chronology are excluded from ICU/composite models but retained for death-specific endpoints.
6c Treatment context	Methods 2.1–2.3; Discussion 4.1–4.3	Partial	Care was not altered by the study. Admission, ICU transfer and death arise under usual local care and capacity. Subsequent treatments, comfort-care and do-not-resuscitate status were not fully characterized or causally modeled.
7 Preparation and quality checks	Methods 2.4-2.5; Supplementary Methods and Tables S4-S6; analysis code	Substantially reported	Visit uniqueness, source linkage, event-time checks, numeric plausibility bounds and explicit text normalization are documented. Measurement availability and vocabulary change are reported; demographic differences in source-error rates and visit-level chronology compliance were not audited.
8a Outcome definitions	Methods 2.3; Supplementary endpoint definitions; extraction code	Reported with limits of ascertainment	Admission uses two documented dispositions. The main secondary outcome is general ICU transfer or index-hospital death within seven days; components use ICU within 72 hours and death within seven or 30 days. Cardiac-unit and admission-duration sensitivities are defined. These are recorded hospital events, not all-cause mortality or adjudicated clinical need.
8b Outcome assessment	Methods 2.3; Supplementary source audit	Reported as algorithmic ascertainment	Outcome labels are extracted from designated source codes and dated hospital events. No independent research clinician adjudication was performed; routine clinical coding and ward designations remain the source basis.
8c Outcome blinding	Methods 2.3; extraction and analysis code	Not a blinded adjudication study	Labels are constructed algorithmically without prediction scores. No blinded clinical outcome-adjudication exercise was performed; algorithmic separation is not described as assessor blinding.

Item and cue	Location	Status	Evidence and remaining limits
9a Predictor selection	Methods 2.2 and 2.5; Table 5; Supplementary sensitivity specification	Reported	Routine initial triage variables and a fixed TF-IDF representation are specified. Core, measurement, missingness and text variants examine information availability. No claim of superiority to contextual language models is made.
9b Predictor measurement and timing	Methods 2.2 and 2.4–2.5; Discussion 4.3; TOPSOR sources	Partial independent verification	The exporter confirms unchanged local locking, initial-KSM selection and the HIS release gate; technical documentation supports distinct initial and retriage records. Assessment identifiers, completion timestamps and edit histories were not exported. No independent row-level chronology or source-log audit was performed.
9c Predictor assessors	Methods 2.2; Discussion 4.3	Partial	Predictors originated in routine first triage assessments. Individual staff qualifications, experience and demographic characteristics were not analyzed; no independent blinded clinician term classification was performed.
10 Sample-size justification	Methods 2.6; Results 3.1, 3.5 and 3.6; Table S12	Partial	All available eligible visits were used, with endpoint-specific event counts, fitted dimensions and apparent/out-of-fold performance reported. No prospective sample-size calculation established adequacy for each model, component or subgroup.
11 Missing data	Methods 2.4; Results 3.1 and 3.5; Table 1 and Table 5; Supplementary missingness summaries	Reported with limitations	Development-only median replacement, indicators, scaling and text-availability exclusions are explicit. Ablations separate selected feature sets, but imputed values can still convey missingness. Missing-data mechanisms and causes of documentation gaps are not established.

Analysis and evaluation

Item and cue	Location	Status	Evidence and remaining limits
12a Data partitioning	Methods 2.1 and 2.6; executed analysis specification	Reported	Fitting uses April–December 2025; chronological evaluation uses January–June 2026. Five-fold patient-grouped development cross-validation refits preprocessing within folds. Prior inspection of 2026 and retrospective revisions are explicitly disclosed.
12b Predictor transformations	Methods 2.4-2.5; Supplementary Methods; analysis code	Reported	Numeric imputation, scaling, categorical ESI and TF-IDF settings are specified. Vocabulary and transformation fitting are restricted to development data, including within cross-validation folds.
12c Model building	Methods 2.5-2.7; Table 5; Supplementary Methods and Tables S9-S12	Substantially reported	Fixed L2 logistic regression settings, four main feature sets, component models, grouped out-of-fold diagnostics and retrospective sensitivity variants are specified. No test-period algorithm or hyperparameter optimization is claimed; no contextual-encoder comparison was performed. The additional analyst-defined pathway masking was specified after coefficient inspection; no blinded clinician-adjudicated removal is claimed.

Item and cue	Location	Status	Evidence and remaining limits
12d Dependence and heterogeneity	Methods 2.6; Results 3.1 and 3.3; Discussion 4.3	Reported within scope	Patient-grouped folds and cluster bootstrap address known repeat visits. Missing linkage creates singleton clusters and leaves unrecognized overlap possible. One hospital cannot estimate geographic heterogeneity.
12e Performance measures	Methods 2.6-2.7; Tables 2-6; Supplementary Methods and Tables S7-S14	Reported	AUROC, AP, calibration, Brier score, ECE, O/E, operating workload and conditional decision curves are defined. Decision actions and threshold ranges are assumptions; net benefit is not observed clinical benefit.
12f Model updating	Methods 2.1 and 2.5 –2.7; analysis code	Not applicable	The revised analysis applies development-fitted predictors unchanged to evaluation. No evaluation-period recalibration or model-updating procedure is included.
12g Prediction calculation	Methods 2.5–2.6; reproducibility code and README	Reported for analysis; restricted fitted model	Executable pipelines define preprocessing and probability calculation with authorized source data. Selected coefficients do not provide the full vocabulary, IDF weights and fitted pipeline needed to execute the exact published predictor.
13 Class imbalance	Methods 2.5-2.6; Supplementary Methods	Reported	No oversampling, synthetic resampling or class weighting was used. AP, event prevalence, PPV and workload supplement AUROC; imbalance alone is not said to inflate AUROC.
14 Fairness methods	Methods 2.6; Discussion 4.3; Supplementary subgroup analyses	Reported with limited scope	Descriptive age, sex and ESI analyses are separated from comprehensive fairness evaluation. No fairness-constrained training, stakeholder-defined acceptable disparity or analysis of additional protected attributes was performed.
15 Outputs and thresholds	Methods 2.6; Results 3.4; Table 4; Supplementary operating-point results	Reported	Probabilities and out-of-fold development thresholds targeting 80%, 90% and 95% sensitivity are defined. Thresholds are carried unchanged into evaluation; actual sensitivity, missed events, PPV and flags per 1000 are reported. No clinical alert threshold is recommended.
16 Differences between periods	Results 3.1 and 3.5; Table 1; Supplementary monthly documentation summaries	Reported with unresolved causes	Cohort characteristics, event rates, linkage, text availability, measurement completeness, text length and vocabulary drift are compared. The causes of the July–August text gap and December measurement change remain unknown.

Ethics open science and public involvement

Item and cue	Location	Status	Evidence and remaining limits
17 Ethics and consent	Declarations: Institutional Review Board and Informed Consent Statements	Partial; public-policy basis reported	The study cites UMP guidance excluding retrospective records studies without care effects from committee submission and the Polish medical-experiment framework. No study-specific approval or exemption decision was obtained. Institutional authorization is stated, but the controller-specific legal basis for processing pseudonymized health data has not been independently documented.

Item and cue	Location	Status	Evidence and remaining limits
18a Funding	Declarations: Funding	Reported	No external research funding is declared. No publication-charge award is asserted.
18b Competing interests	Declarations: Conflicts of Interest and Author Contributions	Reported	The authors declare no conflicts of interest. Approval of the manuscript by all authors has been confirmed.
18c Protocol access	Methods 2.1; Declarations: Protocol and Registration; reproducibility archive	Prospective protocol unavailable	Dated executed analysis specifications and code are supplied. They document retrospective analyses and additions, not a prospectively registered protocol.
18d Study registration	Declarations: Protocol and Registration; Clinical trial number	Reported absence	No prospective registration or public protocol identifier was obtained. Clinical-trial registration is stated as not applicable; no registration number is invented.
18e Data access	Declarations: Data Availability Statement	Reported restrictions	Patient-level records and original narratives are restricted. Institutional access requests may be considered under authorization and data-protection requirements; access is not guaranteed. Aggregate outputs are supplied.
18f Code access	Declarations: Supplementary Materials and Data Availability; reproducibility README	Reported	Executable analysis code and aggregate outputs are supplied. Source records, private predictions, full fitted model objects and vocabularies are excluded from the public archive.
19 Patient and public involvement	Declarations: Patient and Public Involvement	Reported as absent	Patients and the public were not involved in design, conduct, interpretation or dissemination.

Results and fitted model availability

Item and cue	Location	Status	Evidence and remaining limits
20a Participant flow	Figure 1; Results 3.1 and 3.3; Supplementary Tables S1-S3	Reported	Outcome-specific denominators, exclusions, source coverage and the discharge-cutoff sensitivity are described. Fixed-horizon analyses do not require discharge by June end; transfer-out follow-up remains unavailable.
20b Participant characteristics	Table 1; Results 3.1; Supplementary cohort, missingness and follow-up comparisons	Substantially reported	Period-specific demographics, outcomes and predictor availability are supplied, with comparisons by discharge-cutoff eligibility. Treatments, comfort-care status and several sociodemographic dimensions remain unavailable.
20c Period comparison	Table 1; Results 3.1 and 3.5; Supplementary monthly summaries	Reported	Development and chronological evaluation are compared in patient characteristics, event prevalence and documentation completeness; calendar drift is described without attributing an established cause.

Item and cue	Location	Status	Evidence and remaining limits
21 Analysis denominators	Results 3.1–3.6; Tables 1–6; Supplementary analyses; aggregate CSV outputs	Reported	Each endpoint, subgroup, sensitivity and ablation reports its sample and event count. Small-event component fits are skipped and subgroup discrimination is suppressed below ten events or non-events.
22 Access to the fitted predictor	Methods 2.5; Table 6; Table S16; Data Availability; reproducibility README	Incomplete	Selected 50 positive and 50 negative text coefficients per fitted endpoint, structured coefficients, intercepts and numeric preprocessing metadata are supplied. Full vocabulary, IDF and fitted pipelines are not public. Code permits retraining with authorized data, not direct execution of the exact fitted predictor from the shared package alone.
23a Performance and uncertainty	Methods 2.6-2.7; Tables 2-6; Supplementary Methods and Tables S7-S14	Partial	Main and paired AUROC, AP, Brier, ECE and O/E estimates use 500 patient-cluster bootstrap replicates; subgroup analyses use 300. Decision curves have pointwise intervals. Calibration intercept/slope, operating-point counts and several fixed sensitivities remain point estimates. Intervals condition on fitted development models and are not multiplicity-adjusted.
23b Performance heterogeneity	Results 3.3 and 3.5; Table 3; Supplementary subgroup and monthly analyses	Reported within scope	ESI 3 and internally admitted subsets are visible in the main results, with age, sex, ESI and calendar summaries in the supplement. Discrimination is suppressed with fewer than ten events or non-events. No between-hospital performance comparison is available.
24 Updated-model performance	Methods 2.1 and 2.5 –2.7	Not applicable	No evaluation-period updating or recalibration is included in this analysis. Alternative development periods and feature sets are retrospective sensitivity fits, not deployed updated models.

Interpretation and future use

Item and cue	Location	Status	Evidence and remaining limits
25 Interpretation in context	Discussion 4.1–4.2; Results 3.3–3.6; Table 6	Reported	The interpretation concerns local admission decisions and early recorded hospital events. Incremental narrative information is compared with incomplete structured documentation. Component, subgroup, calibration, PPV and workload results qualify whole-cohort ranking; associations are not causal clinical effects.
26 Study limitations	Discussion 4.3; Methods 2.1–2.7	Reported	The manuscript identifies prior data inspection, retrospective specifications, one center, text selection, source-workflow evidence without row-level audit, sparse measurements, documentation change, incomplete linkage and out-of-hospital follow-up, limited events and absent blinded term ratings. Sensitivities do not remove these constraints.
27a Unavailable inputs in use	Methods 2.4; Discussion 4.2	Reported as future requirement	Numeric preprocessing is specified for the evaluated model. Future unavailable or non-finalized narrative inputs should return care to ordinary clinical assessment without model output. An operational abstention or fallback pathway was not validated.

Item and cue	Location	Status	Evidence and remaining limits
27b Interaction with users	Methods 2.7; Discussion 4.1-4.2; Supplementary Methods and Table S15	Reported as hypothetical use	Bed coordination and clinician reassessment are illustrative actions under clinician control. No user interface, live workflow, prospective intervention or prehospital application was tested. Offline single-record runtime excludes production retrieval, network and concurrency.
27c Further work	Discussion 4.2–4.3; Conclusions	Reported	Future work includes independent prospective and geographic evaluation, predefined outcomes, retained source assessment identifiers/timestamps, workload and calibration monitoring, and patient-benefit assessment. No broader clinical readiness is claimed.

Abstract reporting

Item and cue	Location	Status	Evidence and remaining limits
A1 Prediction task and population	Title; Abstract	Reported	The title identifies triage narrative prediction, admission, early critical outcomes and chronological splitting. The abstract specifies adults with ESI 3–5 and available narratives.
A2 Rationale	Abstract: Background and objectives	Reported	The objective is incremental narrative information beyond incompletely recorded structured triage data.
A3 Objective	Abstract: Background and objectives; Methods	Reported	The abstract separates primary admission from the secondary seven-day hospital outcome and identifies retrospective chronological evaluation.
A4 Data source	Abstract: Methods	Reported	Routine electronic records, one center, retrospective chronological splitting and prior dataset inspection are stated.
A5 Eligibility and setting	Abstract: Methods	Reported	Adults, ESI 3–5, available narratives and single-center setting are explicit. The hospital and city are identified in the main methods.
A6 Outcomes and horizons	Abstract: Methods	Reported	Recorded admission includes accepted external transfers. The secondary endpoint is general ICU transfer or index-hospital death within seven days; it is not described as all-cause mortality.
A7 Modeling and evaluation	Abstract: Methods	Partial	Penalized logistic models compare structured predictors with and without text, using 2025 development and 2026 evaluation. Grouped development cross-validation, preprocessing and the additional ESI-only/text-only models are specified in the main methods rather than the abstract.
A8 Performance measures	Abstract: Methods and Results	Reported within abstract scope	AUROC, average precision, an admission calibration slope, a 95% interval, critical-outcome PPV, flags per 1000 and missed events are included. Detailed clustered uncertainty methods are in the main text.

Item and cue	Location	Status	Evidence and remaining limits
A9 Cohort sizes and events	Abstract: Results	Partial	The abstract reports 51,735 development visits with 4,852 admissions and 40,433 evaluation visits with 3,517 admissions. It gives 167 secondary development events and 40,190 evaluation visits with 123 events; the separate secondary development denominator is omitted.
A10 Model inputs	Abstract: Methods	Partial	Initial narrative and incompletely recorded structured predictors are identified. Demographics, arrival mode and measured fields are not enumerated in the abstract; the main methods specify them.
A11 Estimates and intervals	Abstract: Results	Partial	Admission combined-model AUROC 0.835 has a 95% interval of 0.828–0.841. Secondary AUROC 0.913, AP 0.055, calibration and workload are point estimates in the abstract; fuller intervals are in the main tables and supplement.
A12 Interpretation	Abstract: Conclusions	Reported	Conclusions concern local disposition beyond sparse structured records, do not establish physiological deterioration or an actionable alert policy, and require independent temporal verification and external validation.
A13 Registration	Abstract; main Protocol and Registration statement	Reported	The abstract states that no prospective registration was obtained. The main declarations separately state that no public protocol identifier exists and clinical-trial registration is not applicable.

Remaining evidence gaps

No independent visit-level chronology/source-log audit or measured triage-before-orders compliance proportion.

No independent blinded two-clinician term ratings or corresponding adjudicated category-removal model.

No study-specific ethics decision; public committee guidance is cited. Controller-specific processing basis requires institutional confirmation.

No publicly executable complete fitted text model; selected coefficients are not a full model release.

No geographic external cohort, untouched confirmatory evaluation, prospective impact assessment or production-system latency experiment.

Checklist reference

Collins GS, Moons KGM, Dhiman P, et al. TRIPOD+AI statement: updated guidance for reporting clinical prediction models that use regression or machine learning methods. BMJ 2024;385:e078378. https://doi.org/10.1136/bmj-2023-078378. The statement supplies the main and abstract checklist requirements.
